# Polymer Separators and Solid Polymer Electrolytes for Lithium-Metal Batteries

**DOI:** 10.34133/research.1434

**Published:** 2026-09-22

**Authors:** Kangjie Zhou, Yinjing Luo, Hui Zheng, Longsheng Zhang, Tianxi Liu

**Affiliations:** ^1^ Key Laboratory of Synthetic and Biological Colloids, Ministry of Education, School of Chemical and Material Engineering, Jiangnan University, Wuxi 214122, China.; ^2^ School of Materials Science and Engineering, Sun Yat-Sen University, Guangzhou 510275, China.; ^3^ Department of Chemical and Petroleum Engineering, University of Calgary, Calgary T2N 1N4, Canada.

## Abstract

Developing high-performance and high-safety lithium-metal batteries (LMBs) is of importance to meet the growing demands of next-generation energy storage. Leveraging the high structural tunability and chemical versatility of polymer materials, rational design strategies for advanced polymer separators and solid polymer electrolytes have attracted considerable attention, showing great promise for enabling LMBs with enhanced safety and reliability. This review summarizes previously reported design strategies for these key components, highlighting how molecular and microstructural engineering of polymers can address core challenges such as lithium dendrite growth and thermal instability. Recent advancements on the polymer design strategies were systematically analyzed with an emphasis on the underlying mechanisms in regulating ion transport, suppressing dendrite growth, and enhancing operation safety. Finally, current challenges and future perspectives are discussed, which aim at deepening the insight into rational design of polymer separators and solid polymer electrolyte toward further advancement and practical applications of LMBs.

## Introduction

Energy storage devices are currently confronted with a dilemma arising from increasing energy demand and theoretical energy-density limitations of lithium-ion batteries and supercapacitors [1–4]. The energy densities of the lithium-ion batteries are approaching its theoretical upper limit accumulated by amounts of innovative researches, and it is still insufficient to meet the emerging requirements across the fields of energy storage and conversion [5,6]. Lithium-metal batteries (LMBs), employing high theoretical-specific-capacity lithium metal (3,860 mAh/g) as anode, demonstrate extensive potentials compared with the lithium-ion batteries [7–9]. Distinct from the intercalation/deintercalation mechanism of conventional lithium-ion batteries, LMBs realizes charge/discharge process via the repeated lithium deposition/stripping behavior on the surface of metal anode [10]. However, in the LMBs, the metal anode would generate uncontrolled dendritic lithium, owing to multiple factors such as nonuniform Li^+^ distribution and the instability of the solid-electrolyte interface (SEI) layer induced by uneven Li surface [11]. For practical operation, the dendritic lithium growth may markedly influence cycling stability, while dendrite fracture further induces capacity fading and lowers Coulombic efficiency [12,13]. Moreover, the sharp lithium dendrites on anode may penetrate the separator, resulting in internal short circuits and thermal runaway of LMBs [13,14]. The existing safety concerns above are the primary obstacles for the application of LMBs.

Separators or solid-state electrolytes are indispensable components of LMBs, playing critical function of physically isolating the electrodes and regulating ion transport, which can indirectly influence the lithium dendritic growth [10]. However, the commercial polyolefin-based separator has poor compatibility with the electrolyte and dimensional instability under 200 to 250 °C [15–17]. For solid-state electrolytes, poor interface contacts with electrodes, especially at solid-state ceramic electrolytes, induce high interface resistance, thereby reducing the rate performance and lifespan of LMBs [18–20]. Currently, various strategies have emerged to resolve the existing problems above, such as developing advanced functionalized separator or further exploiting solid polymer electrolytes (SPEs) featuring excellent interfacial performance, where the polymer design strategy has been deemed as a general and effective method for developing the separator and SPEs owing to the polymer’s high structural tunability and chemical versatility [21–23]. Focusing on several practical applications of energy storage requirements, amounts of function’s enhancements can be facilely realized through the polymer design strategy, such as end-group graft in high-voltage poly(ethylene oxide) (PEO)-based SPEs or functionalized Li regulation group modification in the dendrite-free separator [24,25]. By exploiting the designable and functionalized polymer materials, it is feasible to improve the shortcomings of commercial separators and develop advanced SPEs [26,27].

**Fig. 1.**
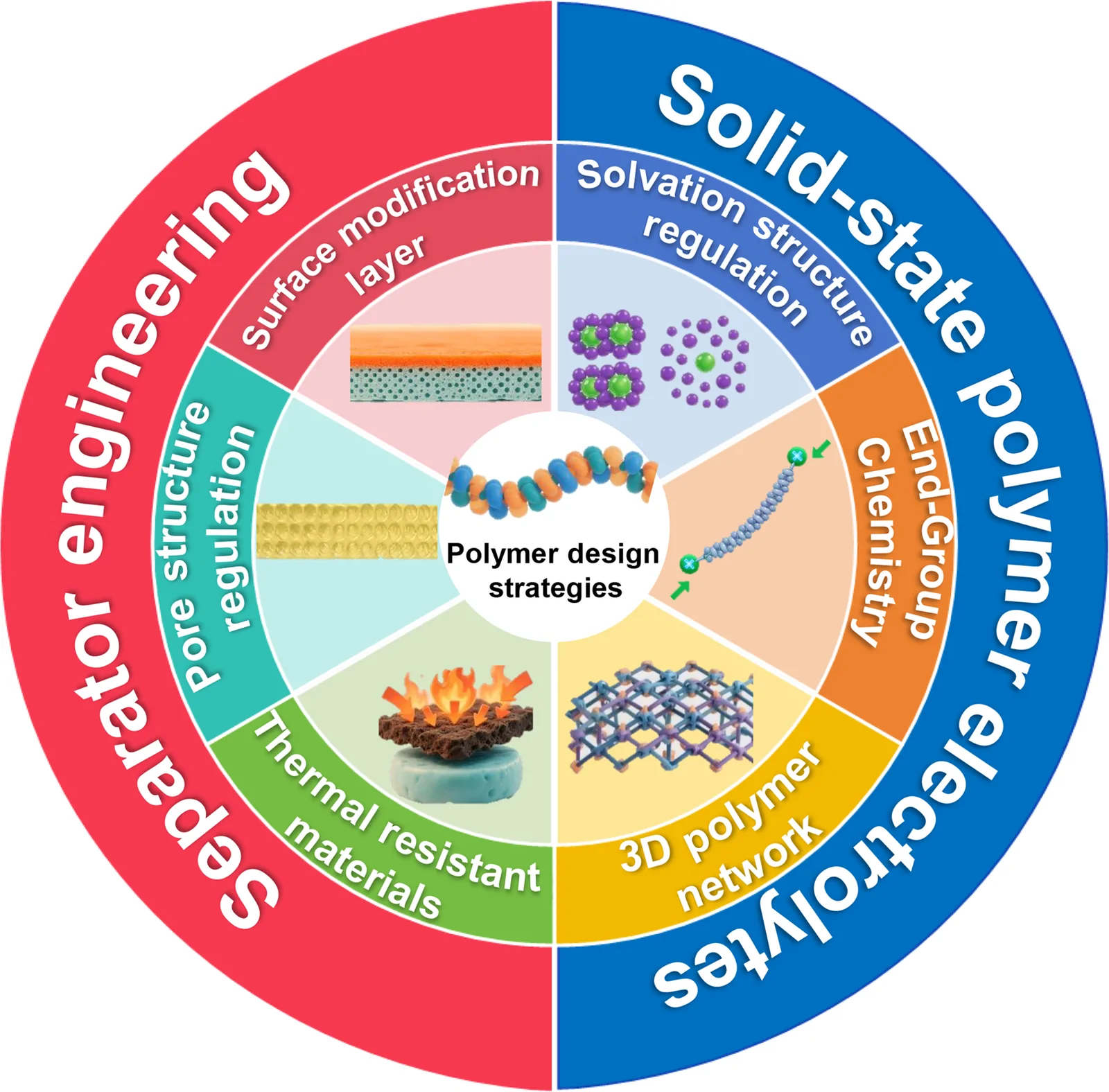
Schematic overview of polymer design strategies for separator engineering and SPEs. The performance and safety of LMBs can be improved through surface modification layer, pore structure regulation, thermal resistant materials, solvation structure regulation, end-group chemistry, and 3D polymer network.

Therefore, this review summarizes the developments of polymer design strategies employed in the advanced separator and solid-state electrolytes based on PEO materials of LMBs. We focus on how change of polymer materials including structure and groups defines the performance of both the separator and SPEs. Introducing rational design and underlying mechanism of polymer design strategies, this review discussed several advances reported recently on function-oriented separators (ion transport regulation, lithium dendrite inhibition, and thermal safety) and performance-oriented SPEs (high ionic conductivity, high voltage, and safety) involving polymer materials (Fig. 1) [28,29]. Specifically, how molecular and microstructural engineering of polymers can address core challenges such as uncontrolled lithium dendrite growth and thermal instability was comprehensively analyzed. Our goal is to connect advances in materials science with existing hazards of LMBs, providing guidelines for developing safe and high-performance LMBs. Finally, we concluded the main research pathway for the advanced separator or PEO-based SPEs through the rational polymer design strategy, providing an outlook for further development of safety and high-performance LMBs.

## Design Strategy of Polymer Separator

Separator is essential elements of LMBs, which allows ion to freely transport inside pore structure of the separator [30,31]. The commercial separator is generally fabricated by polymer materials, such as polyolefin, exhibiting chemical stability and low cost for the battery separator [32,33]. However, its low melting point (≈170 °C) and high surface energy limit the integrated stability under wide temperature and compatibility with the electrolyte, which will induce the safety hazard and poor electrochemical performance [34,35]. Therefore, employing polymer design strategies to develop advanced separators is recognized as an effective method for improving the performance, safety, and efficiency of LMBs. For example, utilizing polar polymers with electrolyte affinity, the separator–electrolyte interface can be effectively improved [36]. For the safety of LMBs, heat-resistant polymers can also be used to prepare high-temperature-resistant safety separators, suitable for safe battery use under wide temperature range conditions [37,38].

Herein, various design strategies for heat-resistant polymers or polar polymers, including grafting, blending, and post-treatment, can be employed to enhance the electrochemical performance while improving the safety of LMBs. According to improvement direction, currently polymer design strategies for separator engineering can be generally divided into 2 categories: (a) enhance electrochemical performance of the separator to improve battery parameters, and (b) optimize the thermal property of the separator to broaden battery application scope. Among them, the polymer’s category, structure, and porosity are precisely regulated to improve the key physical and chemical properties of the as-obtained separator [39–41]. These purposefully designed multifunctional separators unlock great potential for practical use in high-performance and safe LMBs. Building on this framework, the following section will systematically discuss recent advancements in multifunctional polymer separators, highlighting the role of polymer design strategies for improving the performance of LMBs.

## Ion Transport Regulation of Polymer Separator

As an inherently electrically insulating, the separator primarily relies on the electrolyte confined within its micro- or nanometer-scale pores to conduct ions. Consequently, these ion transport behaviors occurring in the separator [ionic conductivity, Li^+^ transference number (*t*_Li+_), and flux uniformity] determined the overall electrochemical performance of LMBs [42–44]. Ions in the electrolyte pass through the electrolyte–separator interface and migrate repeatedly between the positive and negative electrodes. Among ion transport process, interfacial compatibility between the separator and electrolyte stands out as a crucial property that directly determines the interfacial impedance and rate capability of LMBs [45,46]. The polyolefin separators generally show poor interfacial compatibilities for carbonate-based electrolytes, resulting in high interface resistance and slow ion kinetics. Under molecular dynamics simulations, it is identified that the weak interaction between polyethylene (PE) films and ionic solutions does not cause biased distributions of ionic solutions during the diffusion process [47]. By tailoring the separator’s surface properties and chemical functionality, the optimized electrolyte wettability can greatly improve the interfacial compatibility between the separator and the electrolyte, thereby minimizing the interfacial resistance of LMBs and facilitating the rapid ion kinetics.

**Fig. 2:**
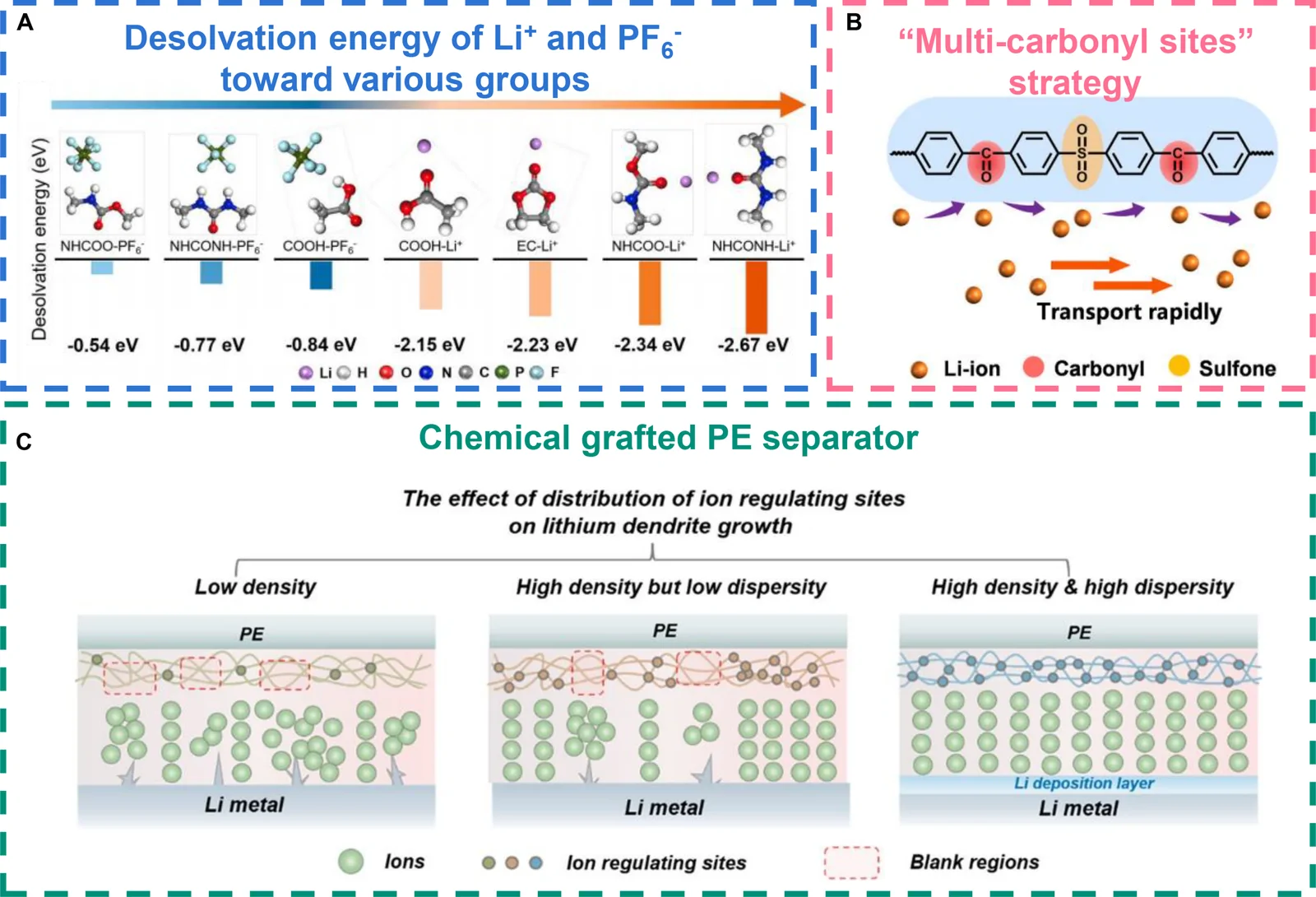
(A) Calculated desolvation energy of -NHCOO, -NHCONH, -COOH, and EC toward Li^+^ and PF_6_^−^ [41]. Adapted with permission of [41]. Copyright 2025, Elsevier B.V. (B) Diagram illustrating the polyether sulfone-based separator design through a “multi-carbonyl sites” strategy [48]. Adapted with permission of [48]. Copyright 2025, Elsevier B.V. (C) Diagram of the ion transport mechanism for the hyperbranched polyamidoamine grafted PE composite separator (HBPA-g-PE) [52]. Adapted with permission of [52]. Copyright 2023, American Chemical Society.

Taking the introduction of specific polar functional groups as an example, the -SO_3_Li group not only possesses a strongly polar sulfonic acid group, capable of forming a strong dipole–dipole interaction with the carbonyl group in carbonate molecules, but also serves as an immobilized anion site, inhibiting anion migration and attracting Li^+^ through electrostatic repulsion effects, thereby constructing an interfacial environment conducive to the rapid transport of Li^+^ at the molecular level. Specifically, the polar -SO_3_Li groups serve as external anion traps, which locally deplete free anions and solvent molecules near the interface, thereby regulating the local solvation structure toward a higher proportion of contact ion pair (CIP) and aggregate (AGG). Groups represented by -COOH and -NH_2_ can form a hydrogen bond network with carbonate solvent molecules and regulate the local solvation structure, greatly reducing the contact angle of the electrolyte on the separator surface and decreasing the formation of solvent-ion coordination. Based on these principles, Fan et al. [41] developed a supramolecular elastomer (ca. 3.2 μm) composed of polycaprolactone diol, tartaric acid, and 3-aminophenylboronic acid, which was coated on polypropylene (PP) separators. The separator-enriched -COOH and -NH_2_ exhibit a high ionic conductivity of 0.79 mS/cm and *t*_Li+_ of 0.64. The calculation of desolvation energy further indicates that the -COOH group contributes to the anchoring of PF_6_^−^, while the desolvation energy of Li^+^ for amino-containing groups is lower than that for -COOH groups (Fig. 2A). Specifically, the urea/carbamate has stronger ability of binding with Li than the solvent, which may participate in the Li^+^ desolvation and compete with solvent molecules for coordination to release more Li^+^. The comprehensive results confirm that the as-obtained coating exhibits excellent Li^+^ regulation capacity and actively participates in Li^+^ solvation sheath formation [41]. Similarly, Liang et al. [48] constructed an enriched carbonyl layer (ca. 3 μm) in a polyether sulfone-based separator as a “multi-carbonyl sites” strategy. These carbonyl sites facilitate the rapid Li^+^ transport (Fig. 2B), achieving a high ionic conductivity of over 1.0 mS/cm.

Although constructing a modified layer directly on the surface of the separator through casting technology can effectively improve the interfacial performance, this strategy still has several limitations in practical applications, especially in terms of thickness control and sacrificing volumetric energy density. When forming a functional modified layer on the separator through casting methods such as scraping, dipping, or spin coating, it is inevitable to introduce an additional coating of a certain thickness. Even with careful control of the viscosity of the slurry and coating parameters, the coating thickness often reaches several micrometers or even higher, which appreciably increases the total thickness. The increase in separator thickness directly leads to a decrease in the proportion of active materials per unit volume of battery cells, resulting in a decrease in the energy density of the battery volume. Previous studies have shown that ceramic coatings with a thickness exceeding 6 μm or a thickness below 1 μm can have adverse effects on the electrochemical and thermal properties of separators [49,50]. Jung et al. [51] further discussed the influence about thickness (2 to 4 μm) of commercial Al_2_O_3_ coating in the separator toward thermal property and electrochemical performance of lithium battery, which could become a guide to coating technology employed in the polymer-based separator. It is suggested that 2 μm of the Al_2_O_3_-coating layer would be suitable for application in lithium battery used for improving energy density and stability. Secondly, many coating materials that can provide excellent interface functionality are chemically incompatible with polyolefin substrates. It is usually necessary to introduce polymer binders to achieve mechanical connection between the coating and the substrate. As an inactive component, the binder may cause a decrease in the porosity of the membrane, partially masking the micropores, thereby increasing the ion transport tortuosity and interfacial resistance. In addition, long-term contact between the binder and organic electrolyte may cause swelling or dissolution, leading to unstable coating structure and performance degradation.

Different from surface coating, grafting techniques usually exhibit superior interfacial compatibility with matrix and represent a powerful tool for functionalizing separator surface, including surface-initiated polymerization, radiation grafting, plasma treatment, and chemical grafting [52]. For instance, Zheng et al. [53] fabricated an electronegative poly(pentafluorophenyl acrylate) polymer brush-grafted PP separator via surface-initiated atom transfer radical polymerization chemistry, where the PP separator was prewetted into solution containing pre-anchoring of initiators, followed by conducting a polymerization to construct one-dimensionally (1D) Li^+^ flux at the nanoscale to enable rapid ion transport and ultra-stable Li deposition. While surface-initiated polymerization yields high grafting density and stable functional layers on the separator, the prerequisite pre-anchoring of initiators renders the process relatively complicated. Sun et al. [54]. prepared an F-modified poly-p-phenylene terephthamide protective layer on the PP separator via fluorine cold plasma treatment. The as-obtained separator exhibited smaller contact angle (18.5°) for the electrolyte than did the PP separator (44.1°), verifying effective surface functionalization and superior electrolyte affinity. The high-voltage LiNi_0.8_Co_0.1_Mn_0.1_O_2_ (NCM811) cell (10.77 mg/cm^2^, electrolyte-to-cathode ratio = 2.3 to 3.0, 200 μm lithium metal) assembled with the as-obtained separator also exhibits superior cycling stability and practical application potential under high cathode load and lean electrolytes. From a scalable manufacturing perspective, straight forward plasma treatment and ultraviolet (UV) irradiation processes facilitate continuous production and demonstrate strong commercial potential in improving the interfacial property of the separator.

Beyond surface chemistry, the inherent pore structure characteristics of the separator, especially the pore size and porosity, play a decisive role in the ion transport dynamics, which is directly related to the migration efficiency and uniformity of Li^+^ between the positive and negative electrodes. When the separator has a suitable micrometer or submicrometer pore size, the electrolyte can rapidly fill the pores under the function of capillarity and shorten the activation time, reducing the bulk resistance and achieving faster transport capable. Increasing the porosity can markedly increase the free volume occupied by the electrolyte in the separator, thus greatly improving the electrolyte absorption and retention capacity. Sufficient liquid absorption not only ensures the improvement of the ionic conductivity but also helps to form a continuous ion transport path, thus playing a key role in improving the rate performance and cycle stability. To obtain the porous polymer separator, numerous fabrication techniques are employed to construct porous structure to ensure efficient ion transport, encompassing dry-stretching, thermal-induced phase separation (TIPS), sacrificial templating, electrospinning, etc. Dry-stretching and TIPS remain the dominant processes for manufacturing the commercial polyolefin (PP and PE) separator [55]. For pore structure, the dry stretching process typically yields separators characterized by crack-like micropores and pronounced anisotropy, mainly employed to prepare the PP separator [56]. In sharp contrast, the TIPS method generates a 3D interconnected porous network while enabling the production of thinner separators compared to the dry method [57].

Notably, with the advantages of high porosity, electrospinning has emerged as a highly attractive approach of preparing the separator with 3D crosslinked pore structure due to its potential for scalable manufacturing. Driven by a high-voltage electric field, the polymer solution droplets are deformed into Taylor cones and subsequently jetted onto a collector, constantly yielding nonwoven nanofibrous membranes, with advantage of scalable production [58]. Crosslinked polymer nanofiber separators fabricated via electrospinning, such as those based on polyacrylonitrile (PAN), polyimide (PI), and polyvinyl alcohol (PVA), possess a 3D crosslinked fibrous network. Characterized by exceptional porosity and an expansive specific surface area, this unique 3D architecture secures superior electrolyte uptake and high ionic conductivity [59–74]. For example, Wang et al. [59] fabricated a composite crosslinked separator by integrating metal-organic framework (MOF) material [MIL-101(Cr)] particles into electrospun PAN nanofibers. This high-porosity framework with Cr metal sites not only realized an impressive ionic conductivity of 3.3 mS/cm but also modulated the transport of non-effective carriers through open metal sites inherent to the MIL-101(Cr) particles. Therefore, superior 3D crosslinked pore structure contributes to enlarge the effect of function-oriented design, which has potential as a framework to develop advanced polymer separators.

Beyond simply maximizing overall ionic conductivity, the ion selectivity of separators is critically related to ion transport kinetics and the rate capability of LMBs. This selectivity can be optimized by tuning the chemical composition of the separator, where Lewis acid-base interactions and electrostatic effects serve as typical principles to simultaneously enhance both ion selectivity and Li^+^ conductivity [22,75]. In the functional design of membranes, Lewis acid sites refer to metal centers or electron-deficient functional groups with empty orbitals and electron pairs. Typical metal centers include Zn^2+^, Ni^2+^, and Al^3+^, while electron-deficient functional groups such as boron- and aluminum-containing groups can also provide empty orbitals. These Lewis acid sites can be used as efficient anion receptors, which can be firmly anchored to the surface of the membrane channel, thus effectively limiting the free migration of anions by coordinating with the lone pair electrons of anions in the electrolyte. On the one hand, this process releases higher amount of free Li^+^ ions. On the other hand, it reduces the anion migration number, substantially improving the Li^+^ transference number, enhancing the dynamics of ion transportation, and improving the rate performance of LMBs. For instance, PE separators grafted with hyperbranched polyamidoamine (HBPA) possess a dense surface distribution of -NH_2_ and amide (-CONH-) groups (Fig. 2C). These electron-rich sites effectively constrain anion migration while facilitating Li^+^ transport through coordination effects, ultimately yielding a high *t*_Li+_ of 0.54 [52]. Alternatively, Wang et al. [75] developed a PE separator coated with a polyvinylidene fluoride (PVDF)/sulfonated poly(ether ether ketone) (sulfonated PEEK)-lithium blend. The intrinsic -SO_3_Li groups restrict anion migration via the electrostatic repulsion, achieving a high *t*_Li+_ of 0.61 while accelerating Li^+^ transport through the desolvation effects. Impressively, the resulting separator enables LMBs with a high LiFePO_4_ (LFP) loading of 10.52 mg/cm^2^ to deliver stable performance for over 200 cycles, exhibiting the importance of ion transport in electrochemical performance. Electrostatic effect employs the selective repulsion or attraction of fixed charge of separator chemical groups toward ions in the electrolyte. A negatively charged transport channel can be formed by introducing a fixed negative charge group into the pore structure of the separator channel, which will electrostatically repel the anions in the electrolyte. At the same time, the charge balance allows Li^+^ to pass through freely, which helps to eliminate the polarization voltage caused by the anion gradient.

The combination of chemical modification and microstructure optimization can overcome the multiple bottlenecks in Li^+^ transport process. For example, in the separator matrix with high-porosity and uniform pores, anion receptors are further anchored or negative charges are fixed by chemical grafting, which ensures the rapid infiltration of the electrolyte and limits the free migration of anions, thus alleviating concentration polarization. In a word, the inherent high structural adjustability and chemical versatility of polymer materials provide a broad design space for the systematic regulation of Li^+^ transport dynamics in LMBs, which has become the core starting point for improving electrochemical performance, reflecting the transformation of polymer separators from passive support components to active functional ion transport control platform.

## Lithium Dendrite Inhibition of Polymer Separator

Although lithium metal possesses high theoretical specific capacity and low electrochemical potential, the uncontrollable growth of lithium dendrites severely impedes the practical application of LMBs, posing critical threats to cycling performance and safety. Fundamentally, lithium dendrite formation predominantly occurs during the charging process. Under ideal conditions, Li^+^ migrating from the cathode through the separator to the anode should deposit uniformly. However, under practical conditions such as high-rate current density, low temperature, or inhomogeneous anode surface, Li^+^ undergoes nonuniform reduction and deposits on localized regions of the anode surface [76]. Under high-rate conditions, the Li^+^ concentration at the electrode interface drastically drops, generating a concentration gradient and a space charge layer, which substantially intensifies the local electric field. Owing to the tip effect induced by local electric field concentration, the incoming Li^+^ preferentially nucleate and accelerate growth at surface protrusions, gradually evolving into sharp lithium dendrites. Ab initio molecular dynamics (AIMD) simulations reveal that, in unmodified carbonate electrolytes, CIP outnumbers solvent-separated ion pair (SSIP). CIP’s heavy solvation and PF_6_^−^ hindrance kinetically impede reductive decomposition and fluoride liberation, whereas SSIP, though rapidly decomposing, is insufficient in proportion [77]. The resulting natural SEI thus turns organic-rich, uneven, and poorly protective, fostering side reactions and dendrite growth and severely compromising battery durability. Nonuniform deposition mechanically disrupts the pristine unideal SEI layer on the anode surface, exposing fresh lithium to continuous side reactions with the electrolyte. The fractured SEI, in turn, further exacerbates nonuniform Li^+^ deposition, forming a vicious cycle. Consequently, the repeated rupture and reformation of the SEI layer, together with dendrite detachment forming dead lithium, continuously consume active lithium and electrolyte, inevitably resulting in low Coulombic efficiency and rapid capacity decay.

**Fig. 3.**
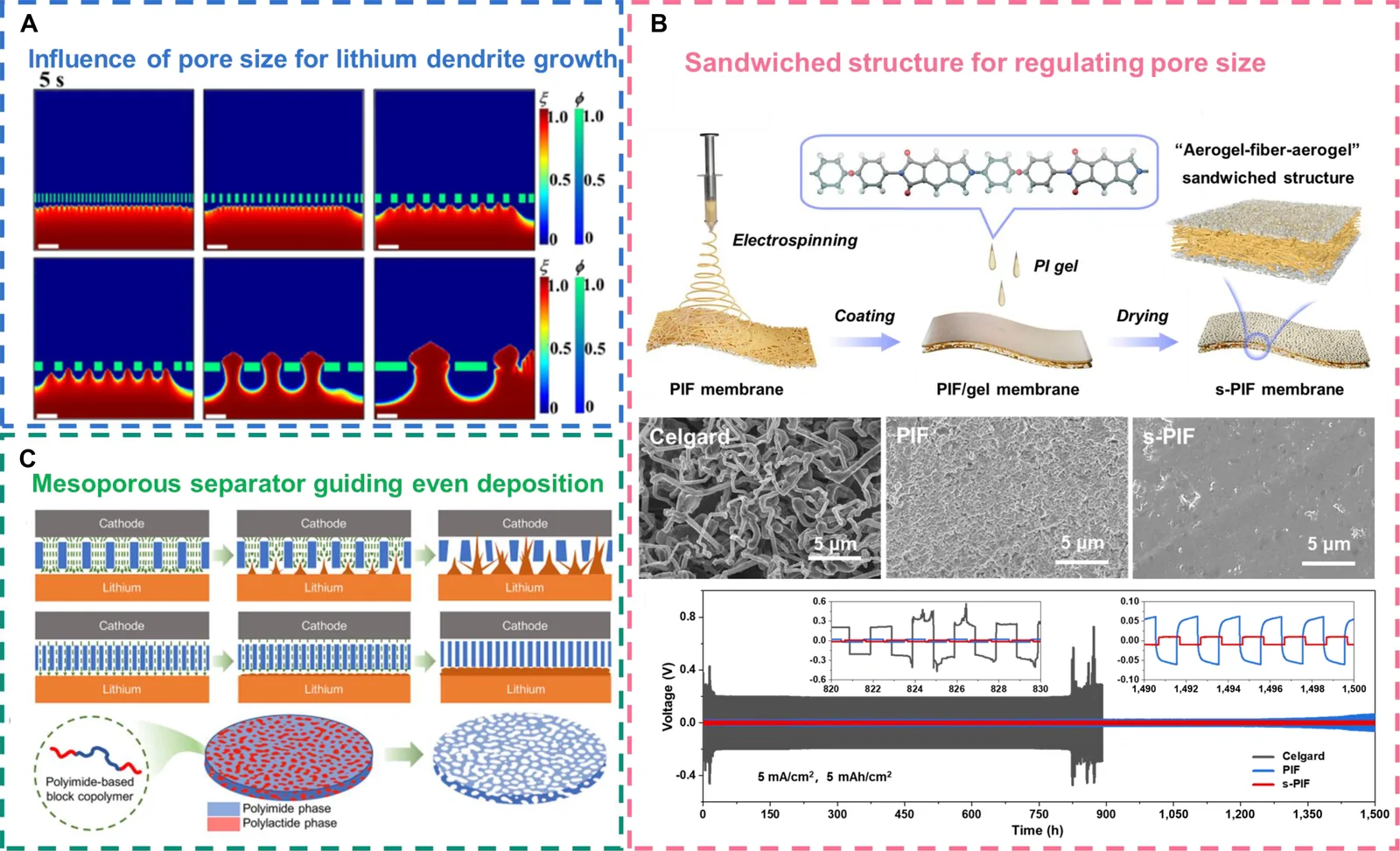
(A) Theoretical calculations illustrating the relationship between pore sizes of the separator and dendritic lithium growth [79]. Adapted with permission of [79]. Copyright 2022, Elsevier B.V. (B) Fabrication process of PI aerogel (PIA)-modified separators (depicted as s-PIF). Voltage–time curves of lithium symmetrical cells with PP, PI nanofiber (PIF) separator, and s-PIF tested in 5 mA/cm^2^ and 5 mAh/cm^2^, and microstructure of cycled Li metal anode [80]. Adapted with permission of [80]. Copyright 2024, Elsevier B.V. (C) Effects of pore size and modulus of separators on dendrite suppression and route to prepare PI-based block copolymer [81]. Adapted with permission of [81]. Copyright 2024, American Chemical Society.

Current polymer separator design strategies for lithium dendrite suppression are primarily focused on 2 directions: one is guiding or homogenizing Li^+^ flux distribution through physical or chemical approaches of the separator, eliminating localized Li^+^ concentration hotspots and inducing uniform lithium metal deposition. For example, in a high-fidelity phase-field model coupled with effective Li^+^ diffusion and conductivity in multi-phase systems, it is confirmed that more uniform coating nanoparticles on the separator are effective for proactive lithium dendrite regulation [78]. The other is enhancing ion selectivity that endows the separator with preferential Li^+^ conduction capability while restricting the free migration of anions through chemical modification, thereby elevating the Li^+^ transference number. As depicted in Fig. 3A, theoretical calculations based on quantum chemistry showed that separators possessing large pore sizes are prone to inducing nonuniform lithium deposition, triggering the risk of Li dendrite penetration [79]. Conversely, a more concentrated and smaller pore size distribution promotes a uniform Li^+^ flux, eventually facilitating a dense and homogeneous lithium anode surface. This result identified that a smaller and uniform pore distribution contribute to facilitate even lithium deposition, eventually inhibiting Li dendrite growth. Therefore, tailoring the pore architecture of polymer separators emerges as an effective approach to inhibit lithium dendrite growth on anode. Employing this pore-regulation strategy, Wang et al. [80] prepared a PI sandwiched separator featuring a PI nanofiber (PIF) middle layer for fast transport and a PI aerogel (PIA) coating with a smaller and uniform pore size of ≈150 nm. By optimizing the pore distribution of the initial PIF separator, the PIA layers enable the stable lithium anode surface at 5 mA/cm^2^ and 5 mAh/cm^2^ for 1,500 h, with little dendritic lithium produced in lithium cells in 200 h (Fig. 3B). Further minimizing pore dimensions, Guo et al. [81] prepared a PLA-b-PI-b-PLA triblock copolymer shown in Fig. 3C, utilizing polylactide (PLA) as a sacrificial template. Through a controlled thermal treatment process to slowly remove the PLA blocks, a mesoporous PI separator with a pore size of approximately 21 nm was obtained. The homogeneous pore size facilitates uniform Li deposition, which effectively validates the physical regulatory role of pore size in controlling lithium deposition. Although the separator with small pore size can inhibit lithium dendrite growth, the reduction of the membrane bulk pore size usually leads to a decrease in porosity, which in turn affects the bulk ion conductivity. Therefore, the pore size design of the separator is a carefully selected trade-off. Comprehensively considering both the Li-ion transmission efficiency and dendrite growth into consideration, Li et al. [81] revealed that a separator porosity within 40% to 50% is suggested for polymer separator design. Remarkably, Sheng et al. [82] found a phenomenon about pore size that a nanostructured photoresist layer (1.41 to 2.77 nm) can partially remove solvent molecules from its primary solvation sheath, suppressing the reactivity between Li^0^ and carbonate electrolyte.

**Fig. 4.**
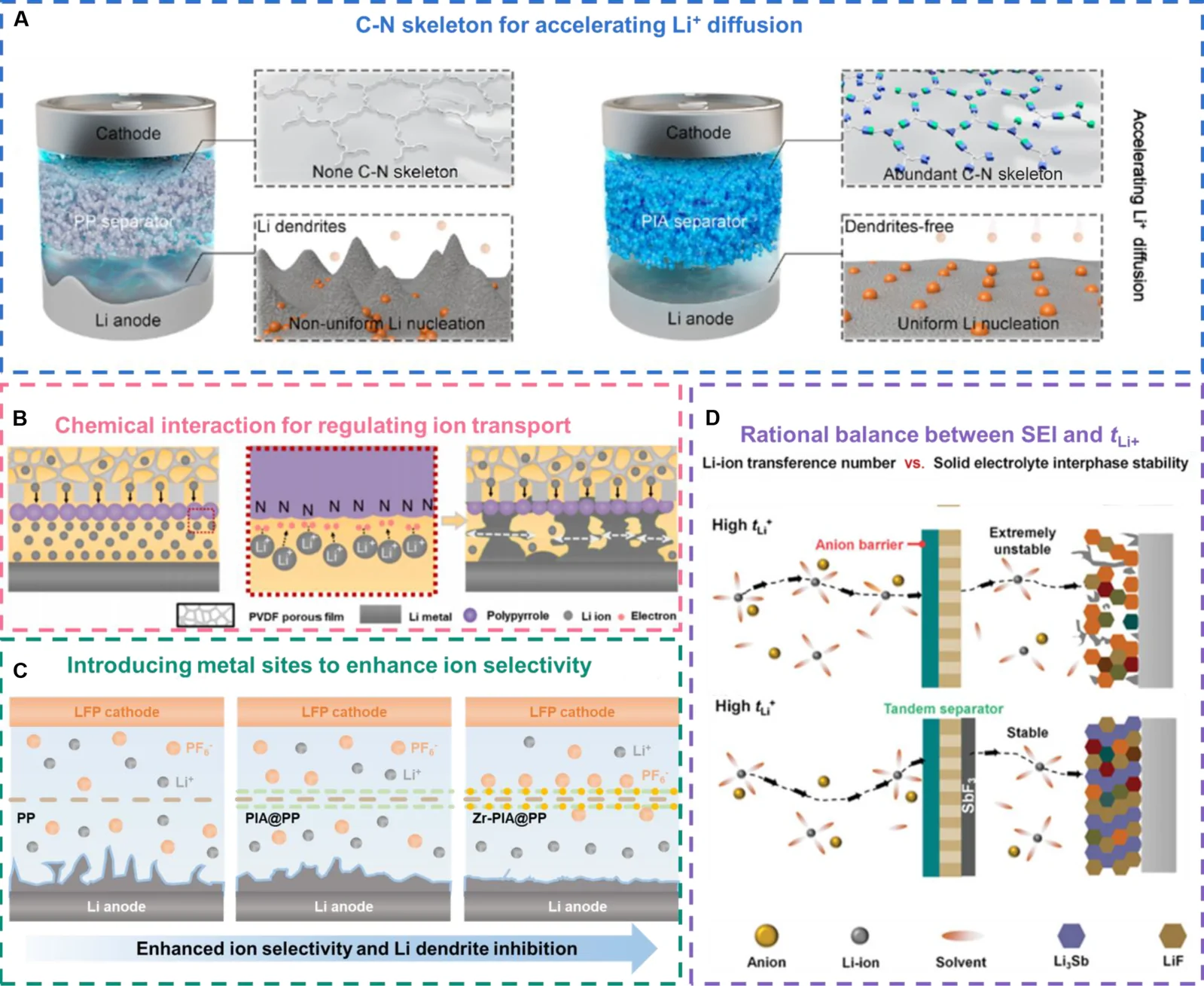
(A) Schematic illustrating that the PIA separator with abundant C–N skeleton can effectively facilitate the uniform Li nucleation [83]. Adapted with permission of [83]. Copyright 2023, National Academy of Sciences. (B) Mechanism of the Janus PVDF/PPy separator for suppressing dendritic lithium [84]. Adapted with permission of [84]. Copyright 2025, Elsevier B.V. (C) Schematic of the Zr-PIA@PP separator facilitating a uniform and selective Li^+^ flux on anode [85]. Adapted with permission of [85]. Copyright 2025, Wiley-VCH GmbH. (D) Schematic illustrating the mechanism of the anion-anchoring separator design and the tandem separator design [90]. Adapted with permission of [90]. Copyright 2023, Wiley-VCH GmbH.

Beyond physical regulation to ion flux via the pore structure optimization, leveraging the chemical interactions between the separator and Li^+^ or their solvation structures in the electrolyte to actively modulate ion transport kinetics and lithium metal nucleation behavior represents another promising strategy. For instance, Ding et al. [83] developed a functional PIA separator featuring precisely tailored nitrogen sites, specifically abundant imine (C=N) and tertiary amine groups. Owing to the differences in electron cloud density distribution of various N-based chemical sites, the PIA separator can attract and repulse Li through electrostatic interactions, facilitating the interfacial Li^+^ diffusion and 2D Li nucleation behavior (Fig. 4A). The high-cathode-loading LFP cells (12.0 mg/cm^2^) with the PIA separator can realize stability cycling over 200 cycles, superior than that with the PP separator. Harnessing similar nitrogen chemistries, Che et al. [84] employed interfacial polymerization to grow a polypyrrole (PPy) layer on one side of a nanoporous PVDF membrane (Fig. 4B), generating a Janus separator. Enriched with nitrogen-containing functional groups (specifically pyrrolic-N), the PPy layer provides active nucleation sites for Li^+^ deposition on the separator surface, effectively guiding the lateral growth of lithium dendrites.

Except directly introducing the chemical group to regulate Li^+^ flux, altering ion selectivity of the separator is another pathway to influence lithium dendrite nucleation through chemical interactions or the polymer structure design. The *t*_Li+_ of the separator is an ion selectivity parameter closely related to the growth of lithium dendrites, which is the fraction of the current carried by Li^+^ in the total current. It is commonly measured by the Bruce–Vincent potentiostatic polarization method, followed by calculating through the following formula:

tLi+=IssΔV−I0R0I0ΔV−IssRss (1)

where *I*_0_ and *I*_ss_ are the initial current and steady state current, respectively. The interface impedance between the electrolyte and electrode was measured before *R*_0_ and after polarization *R*_ss_. Δ*V* is the applied constant potential.

According to the classical sand time model and chazalviel space charge theory, increasing *t*_Li+_ can effectively prolong the initial time of dendrite nucleation *τ* by reducing the number of anion migration, reducing the thickness of the space charge layer at the electrode interface and the local electric field strength. According to the Sand’s time model, the onset time *τ* of lithium dendrite nucleation under galvanostatic conditions is given by:

τ=πDeC024J21−tLi+2 (2)

where *D* is the ion diffusion coefficient, *C*_0_ is the initial salt concentration, *e* is the elementary charge, *J* is the effective current density, and *t*_Li+_ is the Li^+^ transference number. The equation indicates that the nucleation time is inversely proportional to (1 − *t*_Li+_)^2^. As *t*_Li+_ approaches unity, τ theoretically tends to infinity, and dendrite nucleation is substantially postponed.

Consequently, several modifications about the polymer design strategy to increase *t*_Li+_ of separators were developed to suppress lithium dendrite, including the incorporation of metal sites and covalent organic frameworks (COFs) [85–87]. Metal centers typically exhibit strong Lewis acidity and can coordinatively anchor anions in the electrolyte, promoting lithium salt dissociation and releasing additional free Li^+^, thereby attenuating the electrostatic coupling resistance of anions to Li^+^ transport and alleviating the binding interactions between Li^+^ and anion in CIP. Figure 4C illustrates a functionalized separator (Zr-PIA@PP) where single-atom zirconium sites are coordinated on nanoporous PIA coating onto the PP separator. By anchoring Zr sites within the porous aerogel framework to inhibit the free transport of anion in electrolyte, the composite separator achieves a superior *t*_Li+_ (0.52) compared to the commercial PP separator (0.31), effectively delaying dendritic lithium nucleation [85]. Beyond the design of metal sites, the COFs with highly ordered and size-tunable sub-nanometer pores enable precise sieving to allow desolvated or partially solvated Li^+^ to pass, robustly suppressing lithium dendrite growth. Jalees et al. [88] prepared a bifunctional membrane, comprising PI-COFs and polybenzimidazole (PBI), where PBI is used as membrane matrix, while the microstructure and polar functional groups of COFs regulate the flow of Li^+^. The PBI separator-containing COFs exhibit high *t*_Li+_ (0.53) and strong ionic conductivity at room temperature under ideal electronegativity conditions, and achieve relatively uniform lithium deposition.

While high ion selectivity is desired, an excessively high *t*_Li+_ can be counterproductive. Specifically, the over-restriction of anions by the separator results in an “anion-depleted” state at the anode interface, impeding the construction of a robust, inorganic-rich SEI derived from the reductive decomposition of anions. These inorganic constituents are essential for maintaining the mechanical integrity and interfacial passivation capability of the SEI. Excessively suppressing anion migration through the separator design will reduce the free anion concentration and generate the anion-depleted state in the electrolyte–anode interface, rendering organics-dominated SEI produced by solvent molecules for solvent-rich anion-depleted interface fragile and heterogeneous. Therefore, the trade-off between SEI and ion selectivity needs to be carefully considered. Zhou et al. [89] reviewed the several improvement strategies for *t*_Li+_ in liquid electrolytes, where statistical results of most research results showed that a *t*_Li+_ of ≈0.7 is beneficial for delaying the nucleation time of lithium dendrites and does not fall into an anion depleted state. Otherwise, the multiple factor design strategy for both SEI and *t*_Li+_ is also a promising pathway to ensure the long-term cycling for LMBs. To reconcile the competing demands of SEI robustness and high *t*_Li+_, Ding et al. [90] designed an asymmetric separator (Fig. 4D), in which the cathode-facing layer restricts anions to boost Li^+^ transport, while the anode-facing layer reacts with Li to generate beneficial inorganic components like Li_3_Sb and LiF, reconciling the conflict between ion selectivity and SEI quality. The high-cathode-loading NCM811 cells (10 mg/cm^2^) with the asymmetric separator can stabilize cycling over 150 cycles, exhibiting the practical application potential.

Based on the above analysis, the core mechanism of the polymer design to suppress lithium dendrite growth lies in the synergistic optimization of ion transport kinetics and interface stability through molecular engineering and microstructure regulation, thereby breaking the vicious cycle of uneven lithium deposition. Onone hand, by introducing fixed negative charge groups such as sulfonic acid and carboxylic acid groups on polymer chains or pore structure, or anchoring anions in electrolytes using Lewis acid sites such as metal organic frameworks, COFs, and single atom metal sites, *t*_Li+_ can be substantially increased. On the other hand, by constructing uniform and high-porosity nanopores, or optimizing the surface chemistry of the separator with polar functional groups and lithium affinity sites, the Li^+^ flux can be made consistent within the membrane plane, eliminating the preferential nucleation hotspots induced by local Li^+^ enrichment. Therefore, physical and chemical methods through polymer design strategies were effective for guiding the uniform deposition of lithium metal.

## Operation Safety of Polymer Separator

Thermal stability constitutes one of the core parameters ensuring the safe operation of high-energy-density batteries. Traditional polyolefin separators, owing to their limited melting points (approximately 130 to 135 °C for PE and 165 °C for PP), are prone to severe thermal shrinkage and structural collapse under extreme thermal abuse conditions, causing direct physical contact between the cathode and anode and triggering violent internal short circuits. To effectively mitigate these safety risks, advanced design strategies for high-safety polymer separators have attracted extensive attention, among which 3 representative approaches can be identified: flame-retardant separators, separators based on thermally stable polymer matrices, and separators integrated with phase-change materials (PCMs).

**Fig. 5.**
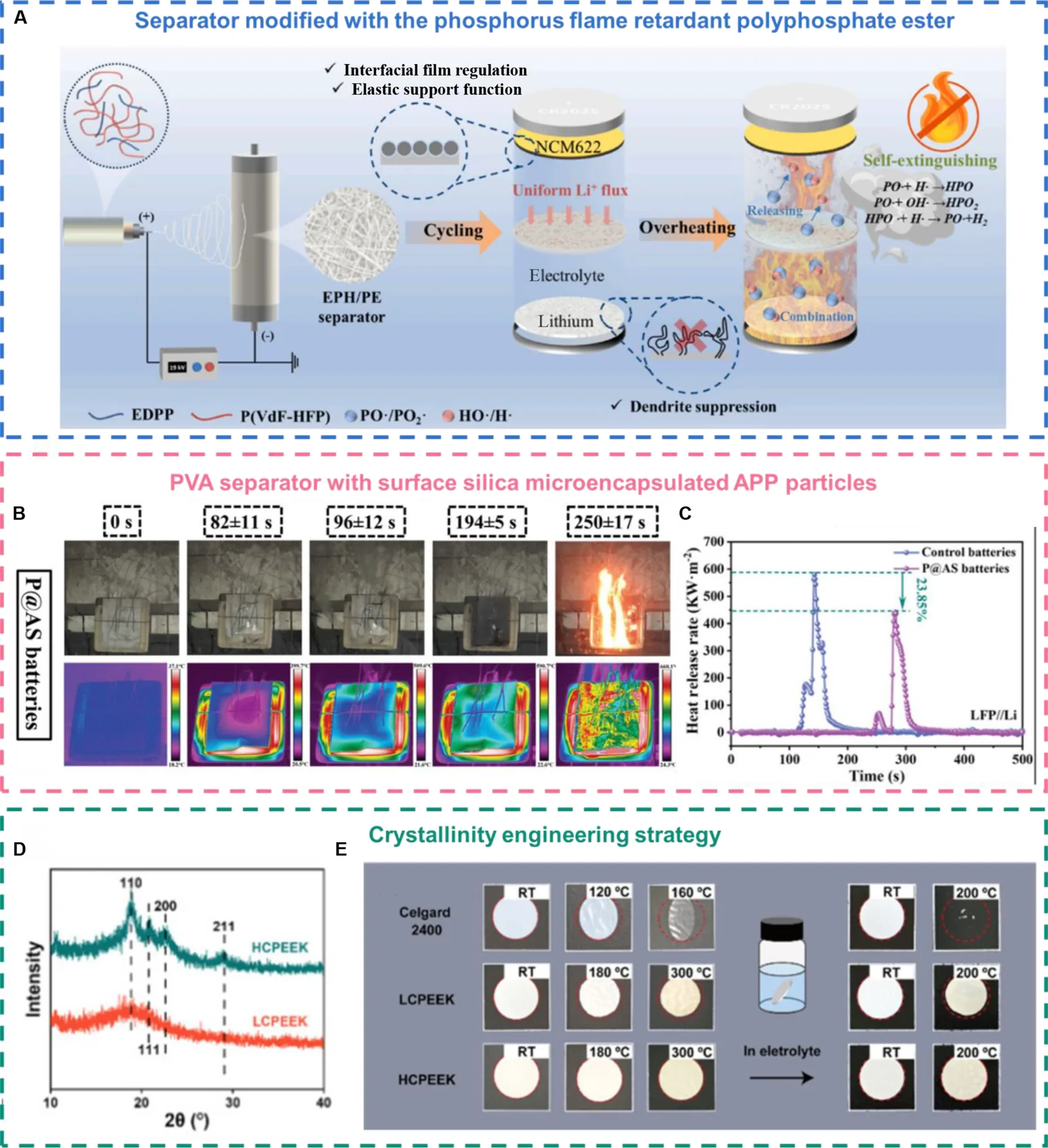
(A) Preparation of the flame-retardant separator via electrospinning [91]. Adapted with permission of [91]. Copyright 2025, Elsevier B.V. (B) Digital photos and (C) HRR curves of pouch cells assembled with the separator, where surface silica (SiO_2_) microencapsulated APP particles anchored in a PVA fiber matrix (P@AS-x) separator under continuous external heating [63]. Adapted with permission of [63]. Copyright 2024, Wiley-VCH GmbH. (D) XRD spectra and (E) thermal stability tests of separators with various crystallinities [98]. Adapted with permission of [98]. Copyright 2025, Elsevier B.V.

Among these strategies, incorporating flame-retardant components into separator matrix offers a robust approach to arrest thermal runaway and mitigate associated catastrophic losses. Fundamentally, the flame-retardant separators suppress electrolyte combustion and the propagation of thermal runaway through the condensed-phase carbonization: Upon ignition of the separator, the flame-retardant additives will decompose at elevated temperatures to generate acidic species, which act as catalysts to facilitate the crosslinking of polymer chains, ultimately converting into a dense carbon-rich layer. The newly formed layer isolates oxygen and internal combustible components, achieving effective flame retardancy and reliable safeguards for LMBs. Representative of this flame-retardant mechanism, phosphorus-based additives like triphenyl phosphate (TPP) and ammonium polyphosphate (APP) decompose upon heating to form a dense phosphate ester layer on the material surface. This layer effectively isolates oxygen and reduces the heat release rate (HRR) of the material. Demonstrating this strategy, Chen et al. [91] reported a separator modified with the phosphorus flame-retardant polyphosphate ester shown in Fig. 5A. The introduction of flame retardant enhances the thermal safety of the as-obtained separator, which achieved a lower self-extinguishing time of 55.73 s/g and better flame-retardant property compared to pure PE separators. Similarly, Liao et al. [63] developed a multifunctional composite separator by anchoring silica microencapsulated APP particles within a PVA fiber matrix. When the battery is burning, this microencapsulated separator can achieve precise release of flame retardant, successfully delayed the ignition time of pouch cells by 138 s (Fig. 5B) and decreased the peak HRR by 23.85% (Fig. 5C). Accurate thermal shutdown is based on the precise release of thermo-sensitive materials during specific temperature. When the temperature exceeds the critical value, the pore size is blocked by thermo-sensitive materials and the ion transport channel is forcibly closed to cut off the exothermic reaction, preventing thermal runaway and improving the safety of LMBs [92,93]. Zheng et al. [94] used coaxial electrospinning technology to encapsulate the flame-retardant TPP in a PVDF shell. As obtained, 1TPP@2PVDF can achieve hot shutdown function at 177 °C and self-extinguishing time of 1.82 s, demonstrating better intelligent protection function. Yu et al. [95] coated poly(melamine terephthalamide) onto the PE separator, which participates in the decomposition of the PE separator and alleviate the degradation rate of PE chain, thereby realizing a wide-temperature-range thermal shutdown. Beyond directly introducing phosphorus-based flame retardants to the polymer, the utilization of the polymer with specific functional groups (e.g., -NH and -CF) offers another effective pathway. Representative polymer materials encompass PI, poly(vinylidene fluoride-co-hexafluoropropylene) (PVDF-HFP), polytetrafluoroethylene (PTFE), and fluorinated acrylate polymers [61,91,96,97]. Specifically, fluorinated polymer materials exhibit excellent affinity for liquid electrolytes, which can enhance the flame-retardant properties of the polymer while also demonstrating excellent interface performance, ultimately enabling the simultaneous strengthening of electrochemical performance and operation safety for LMBs. For instance, Muche et al. [61] reported a 3D cross-linked fluorinated PI/PVDF-HFP nanofiber separator, which exhibited a superior fire-extinguishing capability compared to the PP separator. Furthermore, the polar functionalities, including exposed amide (-NH) and fluoro (-CF) groups, endow the separator with an enhanced electrolyte affinity (a contact angle of 0°) and high ion selectivity, achieving a substantial *t*_Li+_ of 0.7.

Complementary to chemical flame retardancy, enhancing the mechanical strength and intrinsic thermal stability of the separator can effectively suppress the thermal shrinkage and melt rupture of LMBs under high-temperature conditions, where thermal stability directly determines the upper-temperature limit for maintaining dimensional integrity, and mechanical strength safeguards the separator’s resistance to puncture and its ability to preserve internal pore structure, consequently blocking cathode–anode short circuits caused by the separator structural collapse at the source and broadening the safe operating temperature window of the battery. Relevant polymer design strategies typically involve incorporating thermal stability fillers, regulating polymer crystallinity, and constructing hierarchical structures [98–100]. Regarding filler incorporation, most inorganic materials can typically withstand temperatures far exceeding those of polymer matrices while maintaining structural stability, making them ideal candidates for reinforcing polymer matrices. However, the issues of interfacial compatibility and dispersion uniformity between inorganic fillers and polymer matrices, where the former pertains to the physicochemical affinity and bonding strength between filler surfaces and polymer chainswhile the latter concerns the spatial homogeneity of filler distribution within the matrix, constitute a core challenge that must be addressed to realize high-performance composite materials. Zhang et al. [99] developed a composite separator using poly(aryl ether benzimidazole) (OPBI) and functionalized inorganic fillers of boron nitride (BN). Firstly, they carried out precise amino (-NH_2_) functionalization treatment on 2D BN to prepare functionalized BN (f-BN), which solved the compatibility between the inorganic matrix and the polymer. Then, the nonsolvent induced phase separation process was employed to further optimize dispersion of f-BN particles and regulate morphology. Driven by superior thermal stability and low linear thermal expansion coefficient of both components, the resulting separator successfully maintains structural integrity even at 200 °C.

It is highly desired to regulate the intrinsic crystallinity of polymers, which presents another robust approach to bolster thermal resistance without second component. For instance, by using a precise crystallinity engineering strategy, Wang et al. [98] fabricated a semi-crystalline PEEK separator with high crystallinity (denoted as HCPEEK) . As demonstrated by x-ray diffraction patterns, the crystallinity of HCPEEK was systematically regulated via chemical modification and clamping–annealing processes (Fig. 5D). Benefiting from retaining higher crystallinity (22%) compared to its unmodified counterpart, the HCPEEK separator exhibits remarkable thermal stability up to 300 °C (Fig. 5E), which identified the possibility of developing thermal stability materials using a polymer crystallinity engineering strategy. The industry-level LFP/graphite pouch cells (graphite mass loading of 8.4 mg/cm^2^, LFP mass loading of 15.5 mg/cm^2^) can obtain high-capacity retention of 94% at 0.2 C, thus confirming the practical potential of the HCPEEK separator. Furthermore, focusing on the structure design, special structural design strategies exemplified by sandwich and gradient architectures can markedly enhance the comprehensive mechanical properties of separators, particularly in terms of tensile strength, puncture strength, and dimensional stability at elevated temperatures [100,101]. For example, Zhou et al. [100] reported a PI-based sandwiched composite separator incorporating cross-linked APP. This structural design maintains thermal stability at 200 °C through homogeneous PI-based sandwiched structure but also delivers enhanced flame retardance, thereby achieving the lowest total heat release among the tested samples.

As an essential complement to thermal safety strategies such as flame retardancy and thermally stable matrices, integrating PCMs into the separator matrix provides a passive latent-heat-storage-based active thermal buffering defense mechanism through intrinsic solid–liquid phase-change behavior, which can melt and absorb substantial latent heat when the temperature exceeds the phase transition point. Taking paraffin wax as an example, its melting point is approximately 45 °C and its latent heat can reach ≈240 J/g. During heating, this endothermic phase transition efficiently absorbs the heat released by thermal sources, effectively suppressing temperature spikes and delaying or even interrupting the thermal runaway chain reaction. However, the liquid leakage issue of PCMs after melting must be addressed through strategies such as hollow nanofiber encapsulation or microencapsulation. Therefore, these PCMs are typically encapsulated within thermally stable and highly conductive frameworks to minimize adverse interactions between PCMs and electrolyte [102]. For instance, Li et al. [103] prepared a PAN-based separator consisting of a heat-storing electrospun paraffin fibers and a thermally conductive hexagonal BN nanosheet modification layer. The synergistic combination of heat storage and thermal conduction endows the separator with rapid thermal responsiveness and outstanding heat mitigation capability for the in situ thermal management of LMBs (Table 1).

**Table 1.** Summary of the performance comparison between polymer separators

References	Sample	Polymer	*t*_Li+_	Ionic conductivity (mS/cm)	Thermal shrinkage
[41]	PP-CPU	Polyurethane	0.64	0.79	Stability at 160 °C
[48]	PES2K-C	Poly(ether sulfone)	0.79	1.35	Stability at 200 °C
[177]	IISS	PE	N/A	0.464	Stability at 200 °C
[53]	PPFPA-g- Celgard	Poly(pentafluorophenyl acrylate), PP	0.61	0.833	N/A
[54]	F-PPTA@PP	Fluorinated poly(p-phenylene terephthalamide), PP	0.74	1.09	Stability at 165 °C
[59]	PANM	PAN	0.54	3.15	N/A
[52]	HBPA-g-PE	HBPA, PE	0.54	1.13	N/A
[75]	V-40-P/S@PE	PVDF, PEEK, PE	0.61	0.59	N/A
[80]	s-PIF	PI	N/A	1.2	Stability at 250 °C
[81]	Mesoporous PI	PI	N/A	N/A	N/A
[82]	Zr-MOCN@PP	Polymerized Zr-MOC photoresist (Zr-MOCN), PP	0.69	N/A	N/A
[83]	PIA separator	PI	0.78	0.92	Stability at 210 °C
[84]	Janus PVDF/PPy-0.2	PVDF, PPy	N/A	0.732	N/A
[85]	Zr-PIA@PP	Zirconium-coordinated PI, PP	0.52	0.9	Stability at 200 °C
[88]	PBI@PI-COF10	PBI, PI	0.53	1.84	Stability at 200 °C
[90]	Z5P/PE/SBF	Poly dimethyl diallyl ammonium chloride, PE	0.79	0.6	Thickness reduced from 21 to 10 μm at 160 °C
[91]	E2PH/PE	Polyphosphate ester, PVDF-HFP, PE	0.57	0.354	N/A
[63]	P@AS-10	PVA	N/A	1.475	Stability at 150 °C
[94]	1TPP@3PVDF	PVDF	N/A	0.4374	N/A
[95]	PTMs@PE	Poly(terephthaloyl-melamine), PE	0.65	1.21	<5% at 150 °C
[61]	PI/PV	Fluorinated PI, PVDF-HFP	0.7	3.3	Stability at 300 °C
[99]	OPBI@f-BN	OPBI	N/A	1.29	Stability at 200 °C
[98]	HCPEEK	Semi-crystalline PEEK	0.71	0.61	<2% at 300 °C (dry); no shrinkage at 200 °C (wetted)
[100]	PIFAP-2	PI	N/A	1	Stability at 200 °C
[103]	HCHS	PAN	0.49	0.84	Stability at 200 °C

To sum up, the rational design of function-oriented polymer separators is paramount for simultaneously enhancing electrochemical performance and safety of LMBs. By precisely tailoring their chemical composition and micromorphology, polymer-based separators can achieve functional optimization of ionic conductivity, dendrite inhibition, and thermal stability, exhibiting the distinct potential of the polymer design for the development of high-performance, intrinsically safe separators. Fundamentally serving to regulate ion transport and isolating electrodes, polymer separators have undergone a paradigm shift, evolving from passive physical barriers into elaborately designed and safety enhanced materials. Capitalizing on the high structural tunability and chemical versatility of the polymer, advanced structural engineering and functional modifications offer a promising route for further development of high-performance, intrinsically safe separators.

## Design Strategy of Solid Polymer Electrolyte

Similar to separator engineering, exploring solid-state electrolyte is also considered as another pathway to achieve advanced LMBs. Driven by the escalating demand for higher energy-density devices, solid-state electrolyte has emerged as a transformative pathway for next-generation LMBs. The solid-state electrolyte exhibits the likelihood of higher energy density than conditional liquid-electrolyte-based battery and better compatibility with high-energy-density cathode materials, which shows extensive potential in high-energy-density LMBs [104–106].

Beyond the above separator engineering and solid-state electrolytes, gel polymer electrolytes (GPEs) consist of a polymer host swollen with a liquid salt/solvent phase. For GPEs, the liquid-imbibed polymer network usually possesses sufficient polar groups to realize superior electrolyte retention, where solvent and Li salts are filled with the porous framework [107–109]. GPEs are deemed as a pragmatic intermediate offering high room-temperature ionic conductivity and higher energy density, which is closer to commercial applications than SPEs. However, it must be clarified that GPEs retain substantial volatile liquid components. Mechanistically, GPEs are essentially a hybrid architecture that combines liquid-phase ionic conduction with a solid polymeric framework, conceptually overlapping with the separator engineering in liquid systems on one hand and the polymer matrix design in solvent-free SPEs on the other. This work is dedicated to a complementary illustration of polymer engineering in liquid-based systems and SPEs. Therefore, the following sections will introduce the application of the polymer design in solid electrolytes.

Generally, according to materials categories, solid-state electrolytes are classified into solid-state ceramic electrolytes and SPEs. Solid-state ceramic electrolytes achieve conductivity through the rapid migration of specific ions within the crystal lattice, which primarily relies on the directed migration of ions within defects, vacancies, or ordered channels in the crystal structure to complete charge transport [110,111]. Currently, the solid-state electrolyte with the highest room temperature ionic conductivity is sulfide-based electrolyte, which belongs to the ceramic category [112–114]. However, solid-state ceramic materials have severe interfacial problems toward electrodes, thereby inducing high interfacial resistance and poor ion transport, which will greatly hamper the practical application of LMBs [115]. Characterized by superior flexibility and interfacial compatibility with lithium metal anode, SPEs possess the extensive potential for application in high-energy-density LMBs. Serving as the archetype for SPEs, PEO-based polymer electrolytes are the earliest developed materials and have become a prominent research focus.

The ion transport within PEO relies on the coordination between ether oxygen groups in the PEO segments and Li^+^, facilitated by the segmental motion of amorphous regions. Prepared via solution casting or thermal pressing, PEO-based electrolyte membranes can realize good flexibility. However, its practical application in LMBs is heavily impeded by 3 critical bottlenecks: (a) The sluggish ionic conductivity at room temperature (10^−7^ to 10^−8^ S/cm), arising from high crystallinity of PEO, falls far short of practical requirements of LMBs. (b) The intrinsically narrow electrochemical oxidation window (≈3.7 to 3.9 V versus Li^+^/Li) of PEO impedes its compatibility with high-voltage cathode materials. (c) The insufficient mechanical strength of PEO at operating temperatures, which falls far below the mechanical threshold, makes it difficult to construct a physical barrier to effectively inhibit lithium dendrite growth. Validating the efficacy of direct chain modification, advanced polymer design strategies demonstrate immense potential in engineering high-performance and intrinsically safe PEO-based SPEs for LMBs. Addressing the challenges, this section systematically reviews recent modification strategies for PEO-based electrolytes, focusing on advancements utilizing the polymer design strategy to achieve broadened electrochemical window, enhanced ionic conductivity, and operational safety.

## Ion Transport Regulation of Solid Polymer Electrolyte

Different from the ion transport occurring in electrolyte inside the separator, PEO-based SPE itself is a medium for ion conduction, where Li^+^ undergoes a process of “chelation–decomplexion–migration” to achieve leapfrog migration in the PEO matrix with the assistance of the movement of segments. Solvation structure modulation serves as a similar pathway for enhancing ion conductivity in both separators and PEO-based electrolytes. For separators, ionic conductivity hinges on porosity, whereas the ion transport capability of PEO-based SPEs is strongly dependent on the crystallinity of the host matrix. Since ionic conduction is restricted primarily to the amorphous phase, minimizing PEO crystallinity is a pivotal approach to boosting ionic conductivity. Amorphous-rich PEO provides expanded pathways for Li^+^ transport, delivering superior room-temperature ionic conductivity and thus accelerating the rate performance of PEO-based LMBs. Typically, differential scanning calorimetry (DSC) is utilized to assess the crystallinity of PEO-based SPEs, where a diminished melting enthalpy (Δ*H*_m_) during the second heating cycle signifies a reduction in crystalline zones, confirming the success of crystallinity-regulation modifications. The crystallinity structural disruption can be evidenced by a downward shift in the glass transition temperature (*T*_g_). Moreover, XRD characterization can be coupled to probe the crystalline behavior of PEO, opening avenues for maximizing ionic conductivity.

**Fig. 6.**
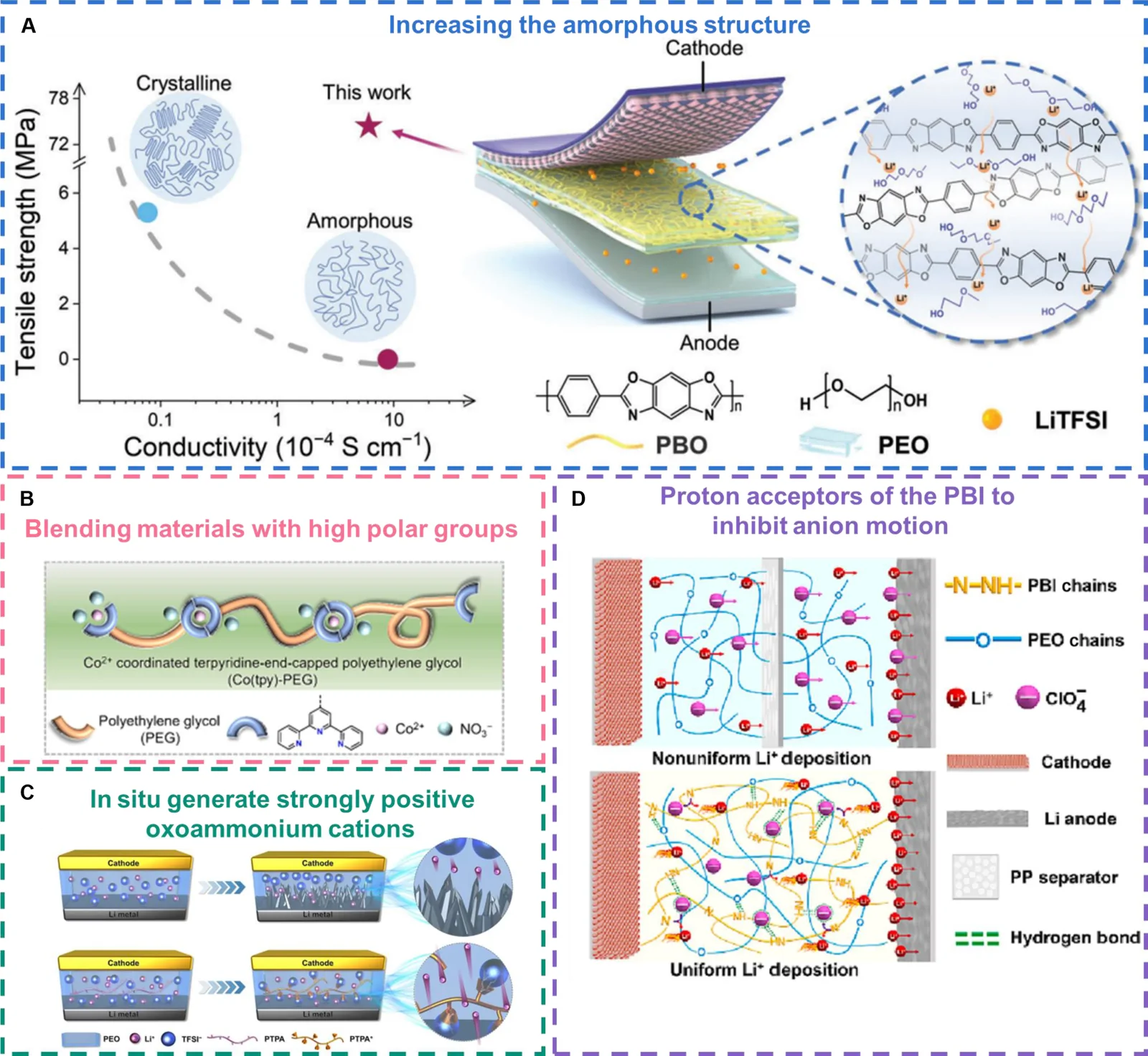
(A) Diagram illustrating the trade-off between ionic conductivity and mechanical strength in PEO/PBO composite SPEs [116]. Adapted with permission of [116]. Copyright 2024, Wiley-VCH GmbH. (B) Diagram illustrating elements of Co(tpy)-PEG [126]. Adapted with permission of [126]. Copyright 2024, Elsevier B.V. (C) Diagram illustrating the function of PTPA on various PEO-based electrolytes (the above of diagram is cells without PTPA and the bottom of that is cells with PTPA) [135]. Adapted with permission of [135]. Copyright 2025, Elsevier B.V. (D) Schematic diagram of Li^+^ transport and Li^+^ deposition behaviors on Li anode surface for the effect of lithium dendrite formation with and without PBI [138]. Adapted with permission of [138]. Copyright 2023, Elsevier B.V.

The introduction of tailored functional groups is widely adopted to further modulate ion transport or interfacial properties, such as intrinsically amorphous polymers or moieties exhibiting lower electron donor numbers than native ether oxygens. Upon the introduction of amorphous polymers, the second component’s polymer chains interpenetrate the PEO chains, disrupting the regular folding of PEO segment into crystal lattices and forcibly maintaining PEO in an amorphous state. Furthermore, the inherently larger free volume of the amorphous secondary component affords expanded spatial freedom for PEO segmental mobility, thereby synergistically accelerating ion transport kinetics. As illustrated In Fig. 6A, the poly(p-phenylene benzobisoxazole) (PBO) nanofiber was introduced into the composite electrolyte as second element to diminish the crystallinity of PEO and to broaden the amorphous domains. The *T*_g_ of PEO decreased from −36 to −40 °C, effectively identifying the reduced crystallinity and resulting in a high ionic conductivity of 6.1 × 10^−4^ S/cm at 60 °C [116].

Fortuin et al. [117] selected PEO and poly(ε-caprolactone) (PCL; with low donor number ester groups) as comparative polymer matrices, introducing typical low donor number groups such as ester groups into the SPE system, and comprehensively verifying the regulatory effect of low donor number groups on the solvation and dissociation behavior of Li^+^ from 3 aspects: coordination environment, charge carrier morphology, and ion transport kinetics. Upon introducing polymers functionalized with low-donor-number moieties (e.g., esters, carbonates, cyano groups, or fluorine atoms), the solvation structure of electrolytes can be altered, weakening the overall environmental binding force on Li^+^ and lowering the dissociation energy barrier [40,118]. Furthermore, blending PEO with polymers featuring highly polar functionalities, such as polypropylene carbonate (PPC) and polyethylene glycol (PEG) derivatives, serves as a robust strategy to boost room-temperature ionic conductivity [119–121]. PPC serves as both a crystallization inhibitor and a plasticizer, aiming to reduce the crystallinity of PEO and thereby enhancing ionic conductivity [122,123]. PEG not only acts as a plasticizer, increasing the free volume of the polymer matrix and providing more migration space for Li^+^ [124,125]. Based on it, Yuan et al. [126] introduced a coordination polymer Co(tpy)-PEG consisting of Co^2+^ terpyridine and PEG (Fig. 6B) into the PEO matrix, where its low crystalline PEG part can efficiently conduct Li. Notably, Co^2+^ competes with Li to interact with anions via Lewis acid-base interactions and enhance dissociation of LiTFSI and weaker interaction between Li and O atoms, leading to the release of more free Li and yielding a high ionic conductivity of 0.22 mS/cm.

Furthermore, employing polymers capable of anchoring anions in the electrolyte is another effective strategy to achieve efficient ion transport [127–129]. In the low-dielectric-constant PEO matrix, lithium salts tend to form uncharged, nonconductive CIPs or even larger ion AGGs [130,131]. When groups with strong Lewis acidity (e.g., boron-containing groups or electron-deficient aromatic rings) are introduced onto the polymer backbone, the backbone acts as anion acceptor [132–134]. According to Le Chatelier’s principle, the strong interaction between the anchoring sites and anions competitively disrupts the electrostatic equilibrium between anions and cations in the electrolyte. This process substantially increases the concentration of free Li^+^ in LMBs, effectively immobilizing anions to promote a higher degree of lithium salt dissociation. A variety of functional materials with capability of anchoring anions in the electrolyte were reported, including poly(2,2,6,6-tetramethyl-1-piperidinyloxy-4-yl acrylate) (PTPA) [135], PVDF-HFP/CeO_2_ nanonetworks [136], covalent organic polymers (COPs) anchored with cationic chains [137], PBI [138], and allyl acetoacetate with *N*,*N*-methylenebisacrylamide [139]. For PTPA, Chen et al. [135] revealed that the radical polymer PTPA (Fig. 6C) generates strongly positive oxoammonium cations (PTPA^+^) in situ, enhancing the release of active Li^+^ through charge-dependent interactions with anions, and providing accelerated Li^+^ transport pathways. For COP anchored with cationic chains, Fang et al. [137] found that the cationic groups on the branches of COPs have the ability to attract more TFSI^−^ and release Li. The free Li passes quickly through the pores of the COPs under the action of ether oxygen chains, which eventually realize a *t*_Li+_ of 0.48. Different from them, Duan et al. [136] innovatively employed oxygen vacancies to assist in anchoring anions. The positively charged oxygen vacancies of Ov-CeO_2_ can effectively increase the Lewis acidity of the PVDF-HFP/Ov-CeO_2_ nanostructured framework, which interacts with the TFSI^–^ and the ether oxygen bonding in the PEO, thus facilitating the mobility of Li^+^. Zou et al. [138] prepared PBI-reinforced PEO-based SPEs by blending PBI, LiClO_4_, and PEO via a solution casting method to form a flexible, self-supporting PBI-PEO-2Li electrolyte (Fig. 6D). The proton acceptors (-N=) on the imidazole rings of the PBI segments form hydrogen bonds with anions to inhibit their motion, which achieved a conductivity of 5.7 × 10^−4^ S/cm and a high *t*_Li+_ of 0.639.

Furthermore, employing porous frameworks or introducing porous additives into the matrix to disrupt the ordered interface of PEO can also effectively suppress crystallinity. High-porosity porous polymer scaffolds substantially enlarge the interfacial area with the PEO matrix, thereby disrupting the local chain ordering and crystalline packing of PEO. Generally, the smaller pore size of the polymer framework indicates higher ionic conductivity. Moreover, molecular dynamics simulations have demonstrated that nanoscale confinement can accelerate the diffusional motion of molecules in SPEs [140], yet this acceleration is counterbalanced when the confining channel is reduced to approximately 2 nm, where the enhanced ion–ion association markedly decreases the population of free charge carriers so that the ionic conductivity does not exhibit a commensurate increase despite the facilitated local dynamics. Poly(2,2,6,6-tetramethylpiperidin-4-yl methacrylate)-wrapped hollow porous SiO_2_ nanospheres were incorporated to create a tailored interface area between surface-modified inorganic filler particles and PEO materials, inhibiting PEO matrix crystallization and establishing rapid Li^+^ transport channels (1.3 × 10^−4^ S/cm at 25 °C) [141]. Inspired by gas foaming technology employed in the polymer porous scaffold field, Li et al. [142] prepared porous PEO-based electrolytes via solvothermal methods. The pore structure obtained via solvent boiling can suppress crystallization of the PEO chain segment and reduce the density of electrolytes, demonstrating the effectiveness in enhancing the ionic conductivity by regulating the PEO interface area.

Besides, the self-supporting ultrathin polymer electrolyte with superior mechanical performance offers another avenue for optimizing ionic conductivity. By reducing the electrolyte thickness, the ion transport pathways are greatly shortened and the volume rate of electrolytes on cells is reduced, thereby accelerating overall ion transport and improving overall portability [143]. This means that more scale-up scope and application, including flexible conformability for wearable devices or higher energy density for storage systems, can be realized. Although harsher requirements were proposed for ultrathin SPEs than conditional SPEs, several methods were developed to further realize the ultrathin SPE-based cells. Liu et al. [144] developed an ultrathin aramid nanofiber (ANF)/PEO-LiTFSI SPE through a novel vacuum filtration method followed by a drying process. Compared to the traditional solution-casted PEO-LiTFSI electrolyte (187 μm), this ANF/PEO-LiTFSI electrolyte fabricated by vacuum filtration features a drastically reduced thickness of 23 μm and forms 3D continuous frameworks without aggregation, delivering a high ionic conductivity of 1.65 mS/cm and exhibiting another strategy to improve electrochemical performance of PEO-based SPEs. Due to insufficient thickness, ultrathin polymer electrolytes still require stringent mechanical properties to achieve self-supporting and molding. Introducing a 3D continuous network to enhance the mechanical properties of electrolytes is an effective way to achieve ultrathin SPEs, such as PVDF-HFP, PTFE, or PE matrix [145–149]. Representative polymer frameworks produced by electrospinning technology exhibits a remarkable role of providing a mechanical support for ultrathin SPEs. Ma et al. [145] employed PAN electrospun film as the matrix, which was filled with PEO and lithium salt ion conductors [lithium bis(trifluoromethanesulfonyl)imide]. The ultrathin SPE with a thickness of 5 μm was successfully prepared, where the unique interface, as well as the good mechanical strength, inhibits lithium dendrites and prevents short circuiting.

## Electrochemical Window of Solid Polymer Electrolyte

Beyond the constraints of sluggish ionic conductivity, unmodified PEO chains typically possess terminal hydroxyl groups (-OH), which render PEO electrolytes highly susceptible to oxidative decomposition at high voltages and unstable at the anode interface. The hydrogen atoms on these end groups are highly reactive and serve as initiation sites for detrimental side reactions in the electrochemical environment. Under high-voltage oxidative environments (typically >3.9 V versus Li/Li^+^), these terminal hydroxyls are highly prone to losing protons and electrons, undergoing oxidative dehydrogenation to generate highly reactive radical species. Rather than merely decaying, these radicals relentlessly attack C–H or C–O bonds along the polymer backbone. This catastrophic degradation culminates in deleterious gas evolution and a drastic surge in interfacial impedance, fundamentally restricting the anodic stability electrochemical window of the electrolyte.

**Fig. 7.**
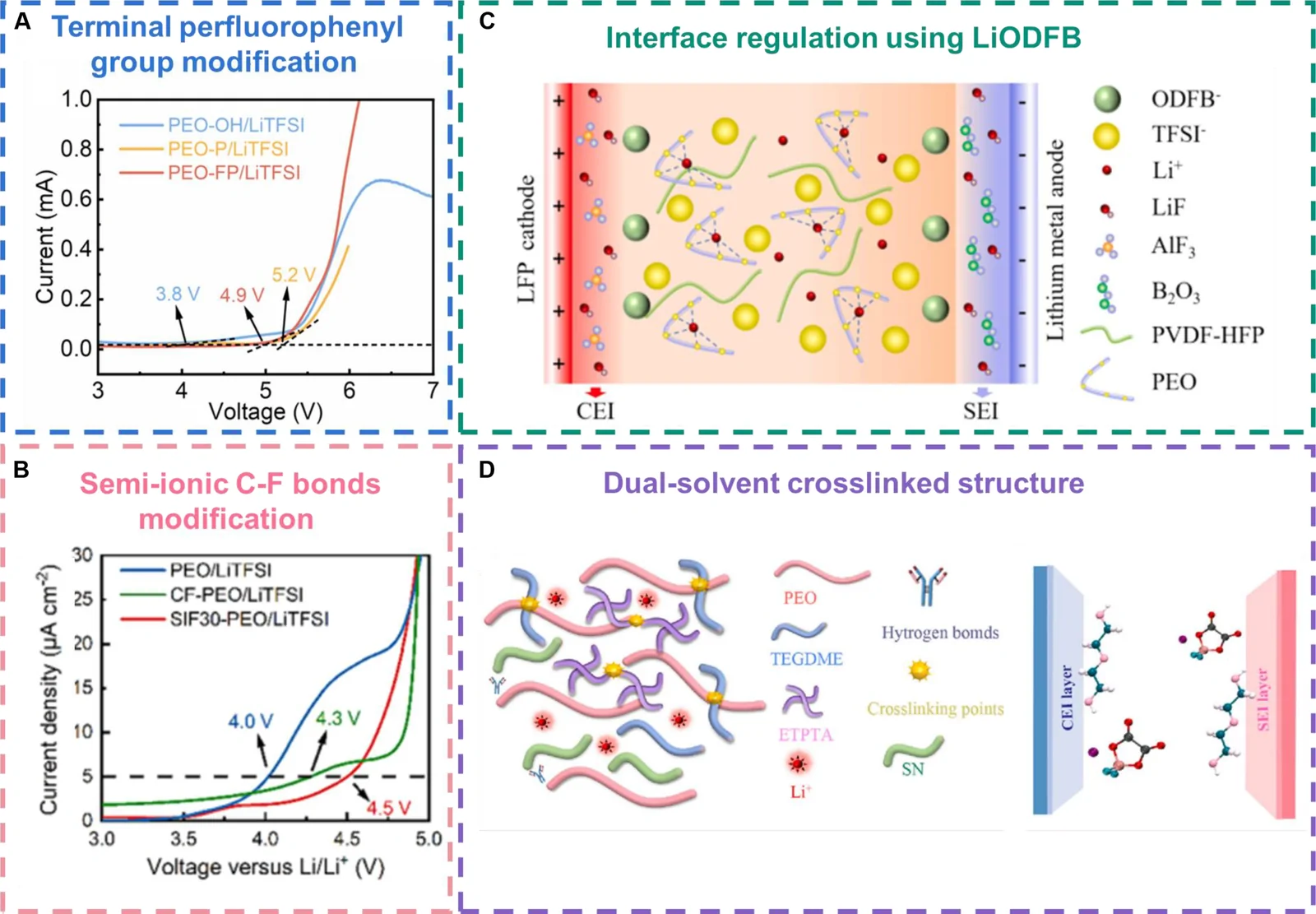
(A) Electrochemical windows of PEO with different terminals [150]. Adapted with permission of [150]. Copyright 2025, American Chemical Society. (B) Electrochemical windows of PEO/LiTFSI, CF-PEO/LiTFSI, and SIF30-PEO/LiTFSI [24]. Adapted with permission of [24]. Copyright 2024, Elsevier B.V. (C) Diagram illustrating that the function of dual lithium salts enhanced composite PEO/PTFE SPEs [151]. Adapted with permission of [151]. Copyright 2025, Elsevier B.V. (D) Schematic illustration of the polymer chains interconnecting and generation of SEI and CEI layer for the dual-solvent PEO-based polymer electrolyte [152]. Adapted with permission of [152]. Copyright 2023, Elsevier B.V.

Therefore, end-group modification, including methylation (forming -OCH_3_) or acylation, serves as a primary method for enhancing the stability of PEO-based electrolytes. For instance, Shao et al. [150] reported 2 novel PEO polymers capped with terminal phenyl (PEO-P) and perfluorophenyl (PEO-FP) groups, synthesized by reacting the terminal hydroxyls of traditional PEO-OH with benzoyl chloride and 2,3,4,5,6-pentafluorobenzoyl chloride, respectively. The results showed that PEO with non-active benzene or pentafluorobenzene end groups exhibited relative stability, expanding the electrochemical window up to 5.2 V (Fig. 7A). The perfluorophenyl group covalently anchors 5 fluorine atoms at the polymer chain end, creating a concentrated electron-withdrawing field and physical barrier, which effectively lowers the highest occupied molecular orbital‌ (HOMO) level, protects the terminal oxygen atoms, and promotes the formation of a dense LiF interfacial layer. In a similar vein, Shen et al. [24] developed a modified PEO featuring semi-ionic C–F (SIF) bonds, which substantially improved electrochemical stability under high voltages and raising the electrochemical window of SIF-PEO-based SPEs from 4.0 V to 4.5 V (Fig. 7B). The LMBs assembled with LiNi_0.5_Co_0.2_Mn_0.3_O_2_ demonstrated high cycling stability at 40 °C (99.79% Coulombic efficiency over 500 cycles at 0.5 C). This work confirms that the strong coordination between ether oxygen atoms and Li^+^ on the traditional PEO chain can be effectively weakened through introduction of semi-ionic C–F bonds featuring electrostatic interactions with HOMO-lowering capability.

Furthermore, the generation of unfavorable by-products at the anode interface is also a major reason for poor electrochemical stability. Uncapped hydroxyl groups undergo irreversible redox reactions upon contact with lithium metal, thereby generating hydrogen gas and lithium salts with poor ionic conductivity (e.g., LiOH or lithium alkoxides). This parasitic interfacial reaction not only consumes active Li^+^ but also forms an uneven, high-impedance SEI on the anode surface, hindering ion transport and inducing nonuniform dendrite growth. To address interfacial issues at the anode, Huang et al. [151] developed composite SPEs by combining PTFE porous membranes with PEO, lithium salts (LiTFSI and LiODFB), and PVDF-HFP (Fig. 7C). The PTFE host–solvent interaction greatly suppresses the activity of free solvent toward lithium anode. Instead, the ODFB^−^ anion in LiODFB possesses superior oxidative stability and preferentially decomposes on the lithium metal anode surface to form a stable SEI layer rich in LiF and B–O compounds, further mitigating side reactions and enhancing electrolyte stability. Xie and Shi [152] designed a dual-solvent PEO-based SPEs, where tetraethylene glycol dimethyl ether (TEGDME) molecules establish a robust UV-cured cross-linked network with PEO (Fig. 7D) while succinonitrile (SN) small molecules engage in strong hydrogen bonding interactions with PEO, integrating LiODFB as an additive. Functioning as plasticizers, TEGDME and SN exhibit exceptional compatibility toward both the Li anode and LiNi_0.6_Mn_0.2_Co_0.2_O_2_ (NCM622) cathode, thus optimizing the electrolyte–electrode interface stability and achieving a high electrochemical window of 5.2 V. The corresponding high-cathode-loading NCM622 cells (10 mg/cm^2^) have a high-capacity retention of 86.6% after 100 cycles, with great potential in practical application.

In summary, the end capping strategy expands the electrochemical window by replacing the active hydroxyl group at the PEO chain end with a strong electron withdrawing group, lowering the HOMO energy level and blocking the oxidative decomposition pathway. Furthermore, the interface engineering strategy constructs a stable electrode/electrolyte interface by prioritizing the decomposition of salt or small molecule plasticizers into a film, further suppressing parasitic reactions and enhancing high-voltage cycling stability. Therefore, both the end-group chemistry and interface engineering exhibit the promising potential in improving the electrochemical window, confirming the effectiveness of polymer design strategies in high-voltage LMBs, which are considered as one of the essential pathways to overcome the high-voltage limitation of PEO-based electrolytes.

## Operation Safety of Solid Polymer Electrolyte

The mechanical integrity of both solid electrolytes and separators serves as the primary physical barrier against lithium dendrite penetration. According to the Monroe–Newman theoretical criterion, the shear modulus of the electrolyte must reach approximately twice that of lithium metal (≈7 GPa) to effectively suppress dendrites [153]. However, the actual shear modulus of linear PEO at operating temperatures is merely on the order of MPa, far below this threshold. Therefore, SPEs with high modulus and superior puncture resistance can effectively suppress dendrite growth at the interface, ensuring excellent cycling stability and operational safety. Current research primarily employs polymer chain engineering strategies to enhance mechanical properties: (a) introducing rigid segments into flexible PEO chains via copolymerization or blending, utilizing microphase separation to form physical crosslinking domains that synergistically optimize mechanical rigidity and ionic conduction [154], and (b) constructing rigid frameworks such as porous skeletons, 3D crosslinked networks, or semi-interpenetrating networks, with PEO serving as the ion-conducting phase and the reinforcing framework as the mechanical support phase, achieving high modulus and exceptional dimensional stability [155]. Polymer electrolytes prepared via these methods typically exhibit excellent mechanical performance and dimensional stability [155–157].

Various reinforcing materials have been reported, including polyamide 6 (PA_6_) [158,159] and polymers of intrinsic microporosity (PIMs) [154]. To improve the mechanical properties of SPEs, Cao et al. [160] introduced hard PA₆ segments into the soft PEO matrix, where the hard PA₆ domains act as physical crosslinking points, thereby maintaining robust mechanical strength, while the soft PEO blocks provide ionic conduction pathways. Furthermore, the abundant amide groups in the PA₆ chains form reversible supramolecular hydrogen bonds, endowing the material with excellent self-healing capability. Fillers not only compensate for the intrinsic softness of flexible PEO but also disrupt its regular crystallinity, achieving simultaneous enhancement of both electrochemical and mechanical performance. Hu and Zhang [154] designed a PEO-based SPEs, with the rigid skeleton of PIM-1 as the hard segments, PEG as the soft segments, and 4,4′-diphenylmethane diisocyanate as the binder. Due to existing excellent mechanical properties of polyurethane hard segments, the high hard skeleton of PIM-1, and the strong hydrogen bonding interactions between polar functional groups (N–H and C=O) and PEO matrix, the designed SPEs have a much shallower displacement under the same pressing force than pure PEO-based SPEs, illustrating that superior mechanical strength tends to restrain the growth of lithium dendrites.

**Fig. 8.**
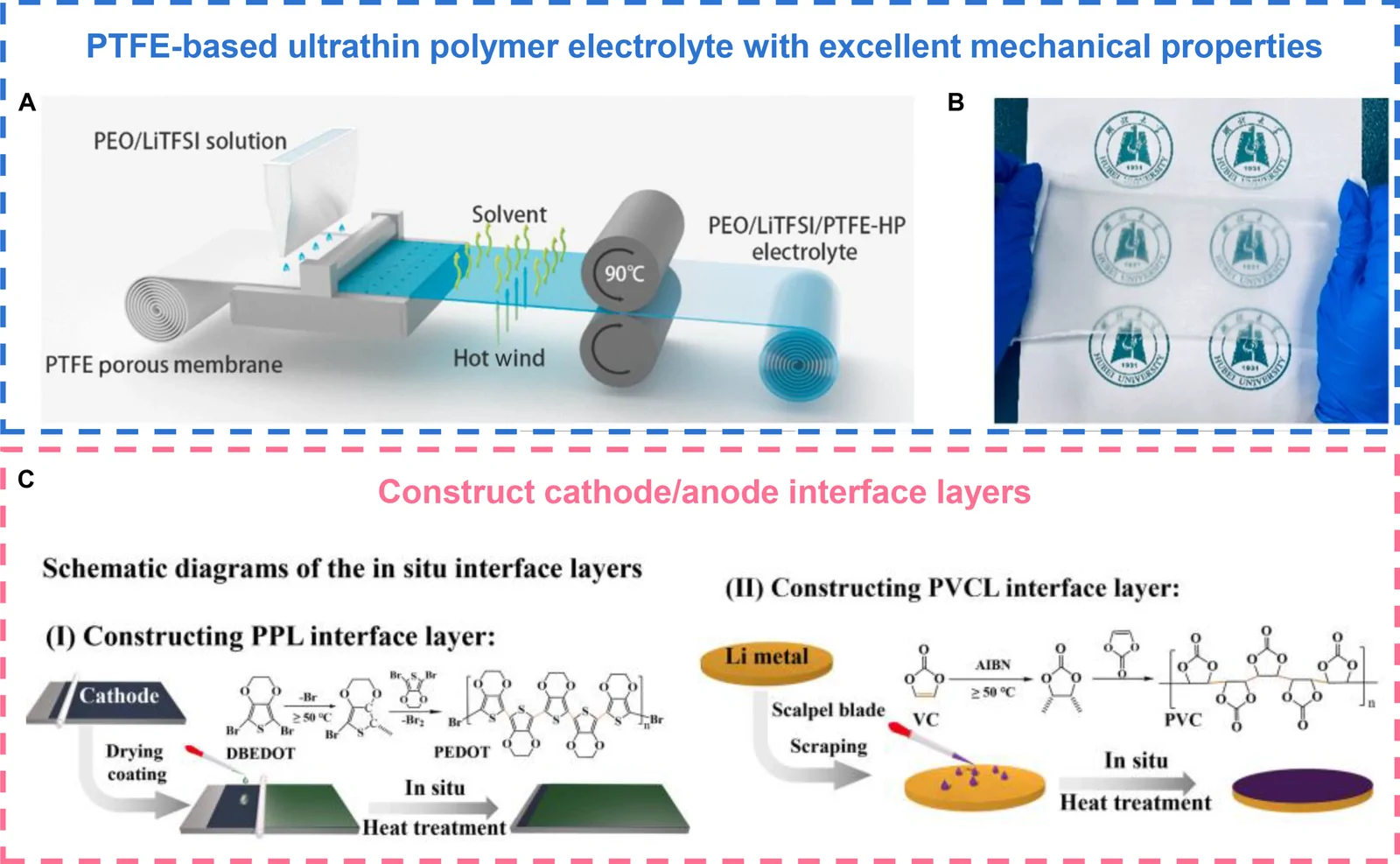
(A) Schematic diagram of preparation process for porous PTFE-based composite polymer electrolytes and (B) its photograph [148]. Adapted with permission of [148]. Copyright 2023, Elsevier B.V. (C) Schematic illustrating the construction strategy of SPEs for both interface layers [164]. Adapted with permission of [164]. Copyright 2023, Elsevier B.V.

Moreover, the 3D cross-linked network typically serves as a filling skeleton for PEO-based electrolytes, effectively enhancing the mechanical property and capability of resisting the dendritic lithium, including electrospun PAN membranes [161] and cellulose membranes [162], which can enhance their mechanical strength and resisting puncture by lithium dendrites. For instance, Liang et al. [148] impregnated PEO/LiTFSI electrolyte into a porous PTFE matrix through a strategic combination of blade-coating and hot-pressing processes (Fig. 8A). The form thickness of the resulting ultrathin composite polymer electrolyte decreased to 6 μm via hot-pressing processes, achieving excellent mechanical properties due to the reinforcing effect of the PTFE matrix (Fig. 8B). The corresponding LFP cells (cathode loading = 7.64 mg/cm^2^) exhibit a quite stable charge–discharge process during cycling. Notably, Sarmad et al. [161] dispersed Li_6.3_La_3_Zr_1.4_Ta_0.6_O_12_‌ particles on the PAN nanofiber membrane using electrospinning, followed by infiltrating a PEO/LiTFSI polymer matrix into the scaffold. The tensile strength of the PEO-PAN electrolyte was appreciably enhanced (5.2 MPa) than that of pure PEO (1.5 MPa). Crucially, the 3D structure of the electrospun PAN membrane acts as a robust physical barrier, effectively blocking dendrite growth and preventing internal short circuits in the battery. However, insufficient adhesion between the skeleton and PEO can lead to increased interfacial voids, hindering ion transport and increasing interfacial resistance. If the content of the skeleton is too low, the enhancement effect is limited. If it is too high, it can lead to a decrease in ionic conductivity. Moreover, in an electrochemical-temperature-mechanical multiphysics field model, Zhang et al. [163] identified that the anisotropy of the bulk membrane will influence the dendrite growth. When the anisotropy strength increases, the growth rate of the dendritic main branch similarly increases progressively. Therefore, the precise design of PEO-based polymer skeletons is also one of the main directions that need to be explored in the future.

Moreover, the interface stability and long-term performance are critical prerequisites for ensuring the operational safety of solid-state LMBs. As a component directly contacting with electrode, SPEs themselves can introduce element or interfacial layers to improve the solvation structure or interfacial reactions, thereby tailoring the interface toward the high-voltage cathode and the high-reactivity anode. Currently, it is known that a robust and uniform SEI is key to increase the lifespan of LMBs. Therefore, precise polymer design strategies for manipulating SEI element are constantly explored. For example, Zheng et al. [164] constructed an asymmetric interfacial layer comprising poly(3,4-ethylenedioxythiophene) facing the cathode and poly(vinyl carbonate) facing the anode (Fig. 8C), yielding stable fast-ion conduction characteristics, with LiF, Li_3_N, and LiBr enriching the lithium metal surface after cycling. This dual-interface strategy provides a reliable pathway to overcome interfacial bottlenecks in solid-state lithium batteries. On the other hand, the detailed mechanism of charge transfer behavior on the formation of SEI is also discovered through the rational polymer design. Liang et al. [165] constructed a porous organic polymer with “charge storage” properties and doped into a polymer composite solid electrolyte to further study the effect of sufficient charge transfer on the decomposition of lithium salts. Further, the LiTFSI decomposition mechanism about the charge transfer process was concluded comprehensively by using charge density difference calculations and AIMD simulation time:

LiNSO2CF32+e−=CF3−+SO2−+LiNSO2CF3− (3)

LiNSO2CF3−+e−=CF3−+LiNSO2− (4)

CF3−+e−=CF2−+F− (5)

Li++F−=LiF (6)

AIMD simulations reveal that SEI formation is governed by a distinct 4-stage pathway induced by consecutive charge transfer. The process initiates with the reductive cleavage of LiTFSI into CF_3_^−^, SO_2_^−^, and LiNSO_2_CF_3_^−^ intermediates, followed by the subsequent reduction of LiNSO_2_CF_3_^−^ into LiNSO_2_^−^. The generated CF_3_^−^ fragments then undergo further electron-driven defluorination to liberate F^−^ ions. Ultimately, the reaction concludes with the combination of Li^+^ and F^−^ to precipitate LiF, thereby driving the phase evolution and structural development of the inorganic SEI layer. Specifically, in the bulk electrolyte, the solvation structure of LiTFSI is influenced by the coordination between polymer chains and Li^+^, as well as the electrostatic interaction between TFSI^−^ and Li^+^, which usually exists in the form of CIPs and AGGs. The TFSI^−^ anions are anchored by the delocalized skeleton via strong electrostatic/Lewis acid-base interactions, which simultaneously weaken the C–S and S–N bonds and extend the Li^+^–TFSI^−^ distance, facilitating the CIP-to-SSIP transition and lowering the lowest unoccupied molecular orbital energy to a pre-polarized state. Upon Li^+^ desolvation at the electrode, the anion’s immobility limited by the polymer results in its interfacial enrichment, thus forming an anion-dense double layer that acts as a substantial feedstock for reductive decomposition. It identified that a porous organic polymer with “charge storage” properties allows LiTFSI to gain enough charge and promotes the dissociation of LiTFSI to take shape a LiF-rich SEI. Impressively, Zhou et al. [166] further optimized SEI components by tailoring the fluorine substitution sites on the benzene rings of porous organic polymers to control LiF concentration in the SEI. The battery exhibited optimal performance when the LiF content was approximately 8.7%. This work provides a crucial strategy for enhancing the performance of PEO-based all-solid-state LMBs via the polymer design and SEI composition control.

**Fig. 9:**
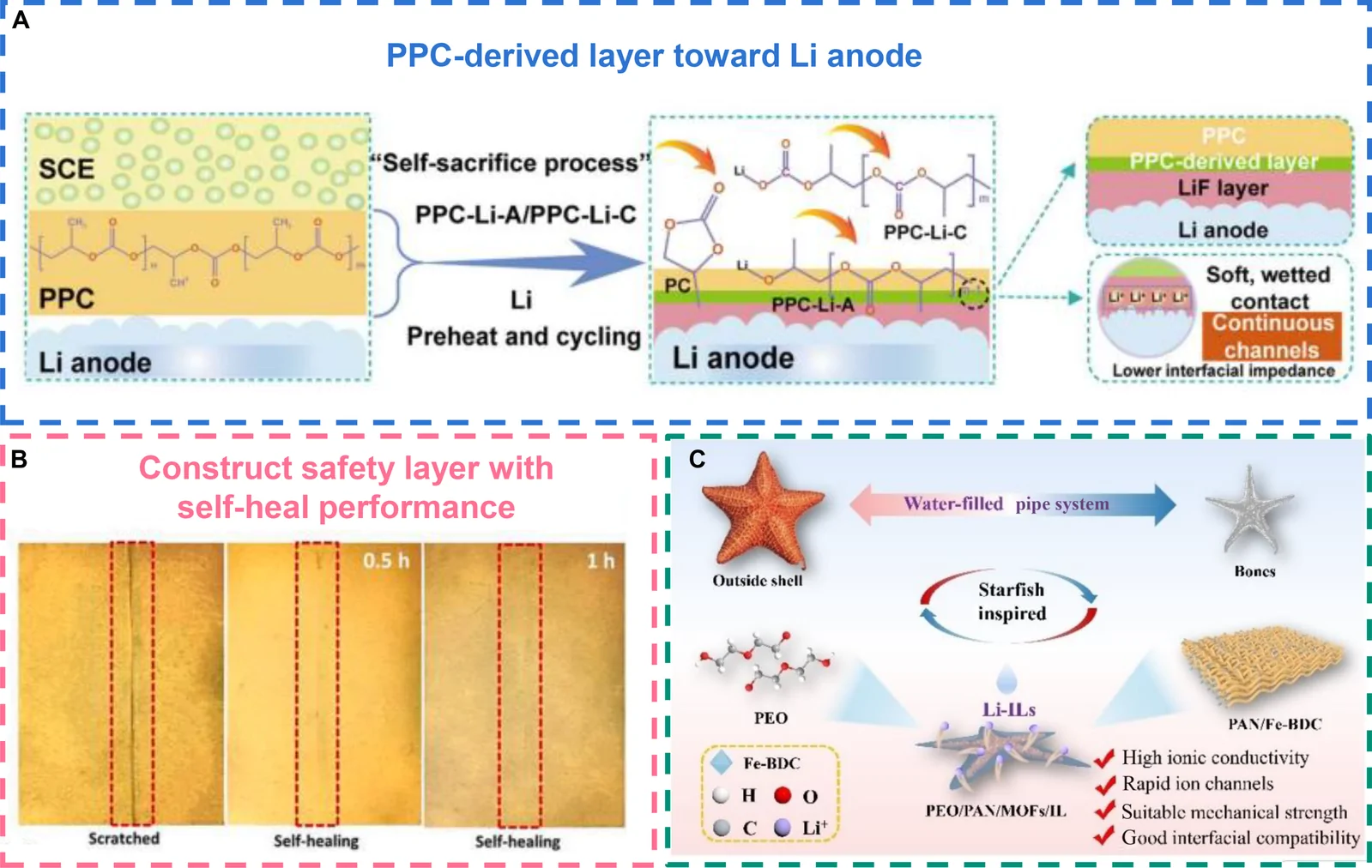
(A) Schematic illustrating the PPC “self-sacrifice” process [5]. Adapted with permission of [5]. Copyright 2024, Royal Society of Chemistry. (B) Digital photos illustrating the self-heal performance of PPC-based SPEs [168]. Adapted with permission of [168]. Copyright 2024, Royal Society of Chemistry. (C) Schematic illustrating the starfish-inspired SPEs and the multifunctional synergistic mechanisms of the PEO/PAN/MOFs/ionic liquid film [174]. Adapted with permission of [174]. Copyright 2025, Wiley-VCH GmbH.

Beyond mechanical and electrochemical stability, the rational polymer design can endow PEO-based SPEs with additional safety-critical functionalities. The exceptional structural versatility of polymers and the multifunctionality of different polymers have endowed SPEs with diversified development routes [167]. Wang et al. [5] proposed an asymmetrical multilayered composite electrolyte design, where a highly flexible PEO buffer layer is positioned against the cathode, while the PPC polymer contacts the anode, effectively minimizing interfacial resistance (Fig. 9A). PEO plays a vital role of contacting with the cathode, while PPC renders decreased interface resistance. The asymmetrical multifunctional design provides the polymer more pathway to improve the existing problem. Gao et al. [168] employed highly reversible ion–dipole interactions to endow composite solid electrolytes with excellent self-healing performance at room temperature (Fig. 9B), with dual ion–dipole interactions between the imidazolium cations in poly(ionic liquid)s blocks and the alkoxy dipoles in PEO and the -CF_3_ dipoles in PVDF-HFP. The high reversibility of ion–dipole interactions endowed SPEs outstanding self-healing property without any external stimulation at ambient temperature. Beyond the dual ion–dipole interactions, a range of physicochemical strategies have been exploited to confer self-healing capability on SPEs, including dynamic imine networks formed via Schiff base reactions [169], in situ alloying with liquid metal [170], reversible imine and disulfide bonds [171], semi-interpenetrating networks based on Diels–Alder dynamic crosslinking [172], and the interaction between 3D amorphous carbon and the PEO matrix [173]. Self-healing electrolytes can repair damage induced by dendrite penetration or mechanical fatigue during cycling, maintaining mechanical integrity and interfacial stability. Additionally, researchers have developed multifunctional “starfish-type” composite polymer electrolytes (PAN/MOFs/ionic liquids). Here, rigid MOF cores are enveloped by a PEO buffering layer to ensure electrode compatibility, while ionic liquids are incorporated in the PAN matrix to minimize interfacial resistance, culminating in a synergy of mechanical and electrochemical enhancements (Fig. 9C) [174].

In summary, through various means such as polymer frameworks and rigid polymer fillers, synchronous enhancement of the mechanical and electrochemical stability of SPEs can be achieved. In addition, the reasonable polymer design, including various groups or multifunctional polymers, endows solid electrolytes based on PEO with safety-critical functions in addition to mechanical and electrochemical stability. These strategies highlight the multifunctionality of polymer engineering in developing advanced and safe PEO-based electrolytes (Table 2) [175,176].

**Table 2.** Summary of the performance comparison between PEO-based SPEs

References	Sample	Polymer	*t*_Li+_	Ionic conductivity (mS/cm)	Tensile strength (MPa)	Electrochemical window (vs. Li/Li^+^)
[116]	PBO/PEO	PEO, PBO	N/A	0.61 (60 °C)	74.4	4.6 V
[117]	PEO/LiTFSI	PEO	0.31	≈0.47 (70 °C)	N/A	N/A
[126]	CPE-PEO/Co(tpy)-PEG	PEO, PEG	0.75	0.22 (60 °C)	>0.9	5.2 V
[135]	PPL	PEO, PTPA	0.57	0.76 (60 °C)	1.05	5.0 V
[137]	CbzTM@C9	PEO	0.48	0.8 (60 °C)	1.26	≈6.3 V
[136]	Ov-CeO_2_-CSE	PEO, PVDF-HFP	0.49	0.176 (30 °C)	7.34	>5.3 V
[138]	PBI-PEO-2Li	PBI, PEO	0.639	0.57 (25 °C)	5.27	4.45 V
[141]	7.5%P@SiO_2_@P-PEO-1/8LiClO4	PEO, PTMPM	0.81	0.13 (20 °C)	N/A	>5 V
[142]	B-PEO/SN/LLZTO	PEO	0.26	0.226 (25 °C)	N/A	N/A
[144]	5ANF25Li	PEO	N/A	0.155 (30 °C)	2.59	4.5 V
[145]	PAN-PEO/LiTFSI	PAN, PEO	N/A	N/A	~0.01	N/A
[150]	PEO-FP/LiTFSI	Perfluorophenyl-terminated PEO	0.49	N/A	N/A	4.9 V
[24]	SIF30-PEO/LiTFSI	Semi-ionic C–F modified PEO	N/A	0.076 (40 °C)	N/A	4.5 V
[151]	CSE-2	PEO, PVDF-HFP, PTFE	0.42	N/A	4.38	5.1 V
[152]	DSPE	PEO	0.79	0.57 (30 °C)	≈1.7	4.6 V
[178]	V-SPE-PEG	PEO, PEGDME	0.33 (60 °C)	0.27 (30 °C)	3.16	4.61 V
[154]	CPE-15% PPU	PIMs, PEG, PEO, polyurethanes	0.68	1.82 (50 °C)	4.1	5.3 V
[148]	PLP-HP	PEO, PTFE	N/A	N/A	63	N/A
[161]	PEO-PAN/LLZTO-8%	PEO, PAN	N/A	0.065 (30 °C)	7.2	5.0 V
[164]	PPL-PEO-PVCL	PEO, PVC, PEDOT, PPL	0.47	0.1025 (25 °C)	N/A	4.6 V
[165]	PEO-POF-1 wt %	PEO, porphyrin-based POF	0.64	0.284	N/A	4.61 V
[166]	CSE-4-FP	Porous organic polymers, PEO	0.155	N/A	N/A	5.0 V
[5]	PEO-SCE-PPC	PEO, PPC	N/A	0.321 (30 °C)	N/A	N/A
[168]	PIBCPE_27	PIL-containing triblock copolymer, PVDF-HFP	0.57	0.37 (25 °C)	5.39	5.0 V
[174]	FAEI	PAN, PEO	0.69	0.437 (25 °C)	29.1	5.34 V

## Conclusion and Perspectives

This review comprehensively discussed recent advancement of polymer design strategy for LMBs, focusing on its application in separators and SPEs. By concluding various methods, functionalized separators and SPEs have demonstrated remarkable effectiveness of the polymer design strategy to accelerate Li^+^ transport and inhibit lithium dendrite growth. However, separators possess mature processing technology but rely on flammable liquid electrolytes and face thermal shrinkage and dendrite penetration risks. SPEs provide intrinsic safety and good interfacial compatibility with lithium metal, yet are constrained by low room-temperature conductivity, modest mechanical properties, and narrow electrochemical windows. The reinforcement in safety is critical for separator engineering whereas the electrochemical performance is lacking for SPEs. In the long term, separators and SPEs should be understood as 2 ends of a single technological spectrum rather than isolated domains. Separators and SPEs are capable of both physical separation and ion transport; thereby, bulk electron-blocking and ion-conducting are the foundations of polymer design strategies. Based on this principle, developing high-performance polymer separator and SPEs via elaborated polymer design strategies can further facilitate the advancement of high-safety LMBs.

Although the polymer design strategy shows great potential in developing the advanced separator or SPEs, the transition from laboratory breakthroughs to practical commercialization faces substantial hinders: First, most experiments merely provide simple verification of the effects of modification methods on electrochemical performance, without delving into the precise experimental design to explore the underlying mechanisms and elucidate specific structure–performance relationships, which provides vague guidance rather than clear inspiration for future researchers in this field. Second, although the SPEs have realized improvements in electrochemical window, their compatibility with next-generation high-voltage cathodes and high-loading anodes still requires further enhancement to compete with liquid counterparts in energy density. Additionally, the mechanical conflict between the rigidity required for dendrite suppression and the flexibility needed for interfacial contact is still a trade-off that single material systems struggle to resolve completely.

Advancements in elaborated polymer design strategies for LMBs can be directed toward comprehensive integration and precise design principles. Research should not merely focus on optimizing a single property, but should concentrate on developing multifunctional systems, including multi-functional, multilayer polymer designs or architectures, thereby meeting various requirements posed by both cathode and anode materials. The trajectory points toward integrated ion-conducting membranes that combine mechanical support, ion transport, and interfacial regulation within a unified architecture. Furthermore, the precise experimental design and in-depth mechanism study can help elucidate the intrinsic pathway to enhance the performance and safety of LMBs. Combining the function-oriented precise integration design with the ever-growing demand can overcome current limitations and pave the way for safe, high-energy-density energy storage solutions via innovative polymer design strategies.
